# Identification of Candidate Genes for Salinity and Anaerobic Tolerance at the Germination Stage in Rice by Genome-Wide Association Analyses

**DOI:** 10.3389/fgene.2022.822516

**Published:** 2022-02-23

**Authors:** Mohammad Rafiqul Islam, Shahzad Amir Naveed, Yue Zhang, Zhikang Li, Xiuqin Zhao, Sajid Fiaz, Fan Zhang, Zhichao Wu, Zhiqing Hu, Binying Fu, Yingyao Shi, Shahid Masood Shah, Jianlong Xu, Wensheng Wang

**Affiliations:** ^1^ Institute of Crop Sciences/National Key Facility for Crop Gene Resources and Genetic Improvement, Chinese Academy of Agricultural Sciences, Beijing, China; ^2^ College of Agronomy, Anhui Agricultural University, Hefei, China; ^3^ Shenzhen Branch, Guangdong Laboratory for Lingnan Modern Agriculture, Agricultural Genomics Institute at Shenzhen, Chinese Academy of Agricultural Sciences, Shenzhen, China; ^4^ Department of Plant Breeding and Genetics, The University of Haripur, Haripur, Pakistan; ^5^ Department of Biotechnology, COMSATS University Islamabad-Abbottabad Campus, Abbottabad, Pakistan; ^6^ National Nanfan Research Institute (Sanya), Chinese Academy of Agricultural Sciences, Sanya, China

**Keywords:** direct seeding, QTL mapping, salinity, anaerobic, GWAS

## Abstract

Multiple stress tolerance at the seed germination stage is crucial for better crop establishment in the direct-seeded rice ecosystem. Therefore, identifying rice genes/quantitative trait loci (QTLs) associated with salinity and anaerobic tolerance at the germination stage is a prerequisite for adaptive breeding. Here, we studied 498 highly diverse rice accessions *Xian* (*Indica*) and *Geng* (*Japonica*), and six traits that are highly associated with salinity and anaerobic tolerance at germination stage were measured. A high-density 2.8M Single Nucleotide Polymorphisms (SNP) genotype map generated from the 3,000 Rice Genomes Project (3KRGP) was used for mapping through a genome-wide association study. In total, 99 loci harboring 117 QTLs were detected in different populations, 54, 21, and 42 of which were associated with anaerobic, salinity, and combined (anaerobic and salinity) stress tolerance. Nineteen QTLs were close to the reported loci for abiotic stress tolerance, whereas two regions on chromosome 4 (*qSGr4a*/*qCL4c*/*qRI4d* and *qAGr4*/*qSGr4b*) and one region on chromosome 10 (*qRI10/qCL10/ qSGr10b/qBM10*) were associated with anaerobic and salinity related traits. Further haplotype analysis detected 25 promising candidates genes significantly associated with the target traits. Two known genes (*OsMT2B* and *OsTPP7*) significantly associated with grain yield and its related traits under saline and anaerobic stress conditions were identified. In this study, we identified the genes involved in auxin efflux (Os09g0491740) and transportation (Os01g0976100), whereas we identified multistress responses gene *OsMT2B* (Os01g0974200) and a major gene *OsTPP7* (Os09g0369400) involved in anaerobic germination and coleoptile elongation on chromosome 9. These promising candidates provide valuable resources for validating potential salt and anaerobic tolerance genes and will facilitate direct-seeded rice breeding for salt and anaerobic tolerance through marker-assisted selection or gene editing.

## 1 Introduction

As the staple food for most Asian people, rice productivity has more than doubled since the “Green Revolution” in 1960s, when the global breeding efforts have been largely focused on improving yields under irrigated lands in Asia and rest of the world (Khush, 2001; Jena and Nissila, 2017). However, abiotic stresses (salinity, drought, cold, heat, etc.) are major obstacles to establishment and yield of rice crops, especially in rainfed areas (Khush, 2005). Rice is normally grown in the semiaquatic environments, and transplanting has been the predominant method in rice planting for decades. However, rice grown areas by direct seeding have been increasing rapidly in recent years because direct seeding offers many advantages such as earlier maturity, reduced water use, planting costs, operational simplicity, and so on, particularly in areas of double rice cropping (Kumar and Ladha, 2011). However, direct seeding of rice (DSR) tends to suffer more from anaerobic stress of flooding, heavy rain, or saline water irrigation at the time of sowing, particularly in fields of small-hold farmers of Asia (Flowers and Yeo, 1995; Hakim et al., 2010). Further, DSR can be exposed to combined stress of salinity in nonanoxic conditions during tidal in lowlands of coastal areas of many Asian countries (Ray et al., 2016; Naveed et al., 2018). Under this kind of scenario, flooding decelerates seed germination and delays seedling establishment (Ismail et al., 2008), whereas shoot growth of rice seedlings can be simultaneously arrested under salt stress due to osmotic stress and high accumulation of Na^+^ in shoot tissues (Munns and Tester, 2008), because rice plants could not maintain their normal energy level under hypoxia (low O_2_ concentration) (Kurniasih et al., 2013). Thus, developing rice varieties with superior ability to germinate under salinity and anaerobic stress is essential for success of DSR.

Rice anaerobic germination tolerance (AGT) is a complex trait and known to be associated with rapid coleoptile elongation at germination stage because elongated coleoptiles allow plants to obtain enough oxygen to meet the required metabolic activity under anaerobic stress. The faster coleoptiles elongate, the more likely rice seedlings can survive under anaerobic stress. Both AGT and salinity germination tolerance (SGT) of rice are complex phenomena controlled by multiple quantitative trait loci (QTLs)/genes, and rice germplasm accessions are known to vary considerably for their AGT and SGT (Ghosal et al., 2020). Identification of important QTLs/ genes and their functional haplotypes (alleles) is an important step to understand the genetic and molecular bases of complex traits (Judson et al., 2002; Niu, 2004). Recently, the genome-wide association studies (GWASs) have emerged as a powerful approach for direct identification of QTLs and candidate genes associated with complex traits in germplasm, particularly in discovering useful germplasm accessions and mining underutilized allele/haplotype combinations for crop improvements (Myles et al., 2009). Association mapping using GWAS has multiple gains over the traditional linkage mapping analysis using biparental populations, including (1) higher mapping resolution, (2) more and significant loci for associated phenotypes, and (3) shortened study time (Yu and Buckler, 2006). In rice, GWAS efforts have been greatly facilitated by the free availability of the seeds and genomic sequences of a core collection of 3,010 rice germplasm accessions (Wang W. et al., 2018). In the past 2 decades, several efforts have been taken to dissect rice AGT and SGT by identifying QTLs/alleles to facilitate functional genomic analyses of the traits and to provide target QTLs/genes for marker-assisted selection breeding (Kamoshita et al., 2002). After screening 8,000 rice accessions, Angaji et al. (2010) reported that a *Xian* (*Indica*) landrace, Khao-Hlan-On, has excellent AGT, which were largely controlled by five putative QTLs (*qAG-1-2*, *qAG-3-1*, *qAG-7-2*, *qAG-9-1*, and *qAG-9-2*). They further fine-mapped a major QTL, *qAG-9-2*, on the long arm of chromosome 9 and determined *OsTPP7* as the most likely candidate gene for *qAG-9-2* (Kretzschmar et al., 2015). Later, Zhang M. et al. (2017) reported several novel genetic loci for AGT in 432 *Xian* (*Indica*) accessions and demonstrated the Hap.2 of one candidate gene (LOC_Os06g03520) associated perfectly with flooding tolerance. Hsu and Tung (2015) reported a strong correlation between the subpopulation groups and five haplotypes of *HXK6* gene; allelic variations of different haplotypes contributes to the phenotypic variation of coleoptile responses to anoxic conditions.

Mishra et al. (2016) reported the different haplotypes of *HKT* genes were associated with varied salt tolerance. Using a panel of 208 rice mini-core accessions, Naveed et al. (2018) identified six loci associated with rice salt tolerance on chromosomes 2, 3, 4, 6, 8, and 12. A similar GWAS by Chadchawan et al. (2017) identified 10 loci/genes on chromosomes 1, 2, 5, 10, 11, and 12 for leaf Na^+^ content in Thai rice. Shi et al. (2017) used 478 rice accessions and identified 11 loci associated with salt tolerance at seed germination stage. Two hundred thirty-two diverse rice accessions were used for photosynthesis measurement under salinity stress and identified two genomic regions on chromosome 5 highly associated with Photosystem II (PSII), and it was reported that chloroplast biogenesis in response to salt stress is important. However, the reported AGT and salt tolerance loci and alleles represent only a small portion of the expected loci/alleles involved in salinity and anaerobic tolerance at the germination stage and few of the identified QTLs were resolved into candidate genes for further validation and application in rice improvement.

Haplotypes can be defined as a linear arrangement of the genes/alleles (Judson et al., 2002) and can be determined through genotypic data (Niu, 2004). Identification of candidate genes and their functional haplotypes (alleles) for QTLs provides important information to determine causal genes and facilitate further validation and application of identified QTL in trait improvement. This has been greatly facilitated with the availability of large numbers of the 3,010 resequenced rice genomes (Wang W. et al., 2018). For example, Naveed et al. (2018) reported 22 candidate genes each with two to four haplotypes for salt tolerance at the germination and seedling stages in rice. Zhang J. et al. (2017) used 211 rice accessions and identified 22 candidate genes each with two to five haplotypes for ferrous iron and zinc toxicity tolerance at the seedling stage. This study reports our recent effort to identify 99 QTLs for AGT, SGT, and anaerobic plus salinity germination tolerance (ASGT), each of which was resolved into relatively few candidate genes’ strong evidence from comprehensive analyses of gene–Coding sequence (CDS) haplotypes (functional alleles). Our results should be helpful to enhance the current knowledge and information on the genetic and molecular bases of both AGT and SGT and to facilitate further validation their functionalities by gene editing and utilization in improving rice AGT and SGT in future marker-aided breeding programs.

## 2 Materials and Methods

### 2.1 Plant Material

A set of 498 accessions of diverse germplasm accessions representing the major global rice-growing regions was selected from 3,000 Rice Genomes Project (3KRGP) as the materials. This pool of germplasm accessions consisted of five subpopulations, including 312 *Xian* (*Indica*), 131 *Geng* (*Japonica*), 14 *Aus*, 15 aromatic/basmati (*Bas*), and 26 admixture (*Adm*). Because the population structure may significantly impact our results in the following QTL identification by GWAS, we divided the 498 accessions into two populations based on the genomic relationships (Wang W. et al., 2018), the whole population with all 498 accessions included, population *Xian* (*Indica*) consisting of 312 *Xian*, 14 *Aus* and 26 *Adm* accessions, and population *Geng* (*Japonica*) consisting of 131 *Geng* and 15 aromatic/basmati (*Bas*) accessions.

### 2.2 Evaluation of the Rice Accessions for Anaerobic and Salinity Germination Tolerance

#### 2.2.1 Evaluation of Anaerobic Germination Tolerance

Ten sterilized seeds of each of the 498 accession were placed in a capped glass tube of 2.5 × 8 cm (diameter × height) filled with distilled water up to 5 cm to submerge the seeds. Glass tubes were incubated in a growth chamber at 27°C ± 1°C, with a 12-h light (approximately 150 μmol m^−2^s^−1^)/12-h dark cycle and 60%–65% moisture without changing water for 7 days. For the control experiment, 10 sterilized seeds were placed on moist filter paper in glass tubes. After 7 days, coleoptile lengths (CLs) of seedlings were measured using a standard glass measuring scale, and the anaerobic response index (RI) was calculated as:

Response index=Coleoptile length (submerged)-Coleoptile length (control)

#### 2.2.2. Evaluation of Salinity Germination Tolerance

Ten sterilized seeds of each accession were placed in two filter papers soaked with 10 ml of 115 mM of sodium chloride in a petri plate (9 cm) during the germination stage to screen salinity tolerance. In the controlled treatment, the same number of seeds per line was placed on a filter paper soaked in 10 ml of distilled water in a petri dish. All petri dishes were incubated under controlled conditions in the growth chamber at a temperature of 27 ± 1°C, with 12 h of light and dark (day/night) and 60%–65% moisture. This experiment was laid out under completely random design with three replications for each accession. The final germination rates (GRs in %) were measured for all germinating seeds 10 days after germination. Total biomass (BM) was taken as dry weight (g) of 5 plants; plants (root and shoot) were dried at 70°C for 3 days and was weighted on a digital high-accuracy balance.

$$\text{Germination }{\left(\text{\%}\right)}^{\;}=\frac{\text{No}\text{. of germinated seeds}}{\text{Total number of seeding seeds}}\text{× }100$$

#### 2.2.3 Evaluation of Anaerobic Plus Salinity Germination Tolerance

To identify promising accessions tolerant to the combined (salt plus anaerobic) stress, 200 accessions tolerant to either salt or anaerobic stress were selected from previous separate screening experiments based on higher germination under stress conditions. Ten sterilized seeds of each of the 200 accession were placed in a capped glass tube of 2.5 × 8 cm (diameter × height) filled with saline solution (65 mM) up to 5 cm to submerge the seeds. Glass tubes were incubated in a growth chamber at 27°C ± 1°C, with a 12-h light period and 60%–65% moisture without changing water for 7 days. For the control experiment, 10 sterilized seeds were placed on moist filter paper in glass tubes. After 7 days, CLs were measured using a standard glass measuring scale and the anaerobic salt RI (ASRI) was calculated as:

ASRI=Coleoptile length (submerged)-Coleoptile length (control)

### 2.3 Data Analyses

Phenotypic data distribution and correlations among measured traits and stress index were computed and plotted by using R statistical software, corrplot package (http://www.R-project.org). The 2.8M SNP genotypic data generated from the 3KRGP were used in this study (Zheng et al., 2015). SNPs with missing rates of more than 20% and minor allele frequency ≤0.05 were removed, leaving a total of 2,760,730 high-quality SNPs used data analyses for the whole population, 1,973,926 SNPs for the *Xian* (*Indica*) population, and 1,371,057 SNPs for the *Geng* (*Japonica*) population in the following GWAS.

### 2.4 Population Structure and Kinship

The 2.8M high-quality SNPs were used to calculate the population structure (Q) and kinship (K) of the 498 rice accessions based on Bayesian clustering and principal components using the software STRUCTURE 2.3.4. The program was run with the following parameters: *k* (1–5), with a variable number of groups; five runs at each *k* value, and for each run, 10,000 burn-in iterations followed by 10,000 MCMC (Markov Chain Monte Carlo) iterations. To calculate the appropriate *K* value, the default method, Centered_IBS, was applied using TASSEL 5.2.23 (Bradbury et al., 2007). The IBS was scaled (1 + F) for the mean diagonal element, where *F* is the inbreeding coefficient for the whole population of 498 accessions. These *Q* and *K* matrix were used for associations and analysis.

Linkage disequilibrium (LD) was measured using squared allele frequency correlations (*r* ^2^) values between pairs of the SNP markers, calculated using the TASSEL 5.2.23 software (Bradbury et al., 2007). To find the relationship between the *r* ^2^ value and physical distance of the identical marker pair, the R package ggplot2 was used to obtain the second-order polynomial curve of *r* ^2^ fitting of the filtered data. The LD decay rate was measured by dropping *r* ^2^ to a half along the chromosomal distance (Huang et al., 2010).

### 2.5 QTLs and Candidate Gene Identification by GWAS and Haplotype Analyses

The linear mixed-effects model was applied to determine the association between each SNP and the measured traits (Zhang et al., 2010) by using an efficient mixed-model analysis with the EMMA expedited (EMMAX) software (Kang et al., 2010) to determine genetic similarities among accessions. The effective number of independent markers (N) was calculated using the GEC software (Tumino et al., 2016). As mentioned previously, all rice accessions used in this study were divided into three panel populations, the whole (*Xian* + *Geng* + *Adm*, *n* = 498), *Xian* (Indica) (*n* = 326), and *Geng* (*Japonica*) (*n* = 146) populations. It is well-known that the smaller the population size is, the less the power is in detecting QTLs by GWAS. Based on population sizes, two thresholds of *p* = 1.0 × 10^−5^ and 1.0 × 10^−6^ were used to claim significant SNP-trait associations in the *Geng* (*Japonica*) population, *Xian* (*Indica*) and whole populations, respectively, determined by the total number of SNPs (0.05/N) in GWAS. Based on the LD estimates (*r* ^2^) of 200, 170, and 220 kb of the whole, *Xian* (*Indica*), and *Geng* (*Japonica*) populations (Wang W. et al., 2018), any two or more significant SNPs in <200-kb distances were considered as a single QTLs. Genes inside the QTLs with *p* = 1.0 × 10^−4^ were used for GO (Gene Ontology) enrichment, and the group with false discovery rate ≤0.05 was regarded as significantly enriched. The Pearson correlation coefficient was calculated in R software version 3.5.0, and the network was visualized using Cytoscape version 3.6.1.

Identification of candidate genes for important QTLs was achieved by determining important QTLs for the measured traits and then by identifying candidate genes for important QTLs by bioinformatics and haplotype analyses. From those QTLs identified by GWAS, anyone that met at least two of the following three criteria was considered as important QTL: (1) having large phenotypic effects with >10% of the total trait variance and (2) the LOD peaks of significant SNPs mapped to the same location (∼1 Mb) of a fine mapped QTL/cloned gene of functional relevance, (3) QTL region–containing nonsynonymous SNPs in their CDS −log10 (*p* < 10^−4^). Then, three steps were used to identify candidate genes for important QTL regions by (1) searching all genes in the target region of important QTL-containing nonsynonymous SNPs in their CDS regions based on corresponding reference genomes (Minghui 63) used as the *Xian* (*Indica*) reference genome and Nipponbare as the *Geng* (*Japonica*) reference genome); (2) the presence of major gene CDS haplotypes (gcHaps) in more than 10 accessions that showed statistically significant differences between the major haplotypes of selected candidate genes for the associated trait; and (3) for each of the most likely candidate genes, a haplotype network in the 3KRGP was constructed using all nonsynonymous SNPs downloaded from the Rice SNP-Seek Database (Alexandrov et al., 2015) within its coding sequence regions using the RFGB v2.0 (Wang et al., 2020) and the pegas package in R. Haplotypes present in at least 150 rice accessions in 3KRGP were used for comparing. The mean trait value of each haplotype was compared using one-way analysis of variance (ANOVA) followed by Duncan new multiple range tests (*p *< 0.05) with the agricolae package in R. Then, each of the candidate genes was interpreted according to its population organization and evolutionary relationships of their major gcHaps in the 3KRGP (Supplementary Figure S1).

## 3 Results

### 3.1 Phenotypic Variation and Trait Correlations

The 498 accessions showed huge variation for the measured AGT and SGT traits (Table 1). On average, the *Geng* (*Japonica*) population showed significantly higher values than the *Xian* (*Indica*) population for most the AGT and SGT traits. ANOVA showed that the genotypic differences among the accessions explained 97.2%, 32%, and 97.5% of the total phenotypic variances of GR under anaerobic condition (AGr), CL, and RI contributing to AGT (Supplementary Table S1), and 97.4%, 90.3%, and 97.1% of the total phenotypic variances of GR under salinity condition (SGr), and BM contributing to SGT, respectively (Supplementary Table S1). As expected, highly positive correlation was observed between the three AGT traits, with *r* = 0.82 between AGr and CL, 0.82 between AGr and RI, and 0.99 between CL and RI, indicating that CL was the primary determinant of AGT. For the SGT traits, moderately positive correlations were observed between SGr and BM (*r* = 0.31), (Supplementary Figure S2). Based on the screening result, we were able to identify 53 promising accessions, including 33 AGT accessions (21 *Geng* and 11 *Xian* accessions) from 14 countries, which had mean AGr >79%. There were 14 SGT accessions (5 *Geng* and 9 *Xian* lines) from 12 countries that had mean SGr >76% (Supplementary Table S2). There were 17 RIs under anaerobic and salinity condition (ASRI) accessions (16 *Geng* and 1 *Xian*) from eight countries that had mean ASRI >2.9 cm. Ten of these accessions were promising under more than one stress. Eight accessions showed excellent performance under AGT and ASRI, and one accession (IRIS_313-10710) showed excellent germination under AGT and SGT stresses. One accession (B068) showed excellent performance under three stresses: AGT, SGT, and ASRI (Supplementary Table S2). These accessions would be valuable sources of genetic variation for genetic/molecular dissection and improvement of rice AGT and SGT in the future.

**TABLE 1:** Performances of salt tolerance related traits measured at germination under salinity and anaerobic stresses.

Traits	*Geng* (*Japonica*)			*Xian* (*Indica*)		
	Range	Mean ± SD	CV (%)	Range	Mean ± SD	CV (%)
Salt germination rate (SGr, %)	0.00–96.0	32.1 ± 29.2	38.9	0.00–90.0	25.4 ± 18.9	33.2
Biomass (BM, g*100)	0.53–1.99	1.06 ± 0.30	22.7	0.25–2.20	1.10 ± 0.35	25.4
Anaerobic germination rate (AGr, %)	15.0–96.0	47.5 ± 20.0	41.0	0.00–86.0	26.1 ± 26.5	36.3
Coleoptile length (CL, cm)	1.23–5.23	3.30 ± 0.76	19.8	0.93–4.00	2.50 ± 0.33	22.7
Response index (RI)	0.33–4.47	2.69 ± 0.76	24.8	0.53–3.42	1.90 ± 0.50	21.5
Anaerobic salt response index (ASRI)	0.69–2.53	1.68 ± 0.79	40.2	0.12–2.01	1.02 ± 0.82	38.1

### 3.2 Population Structure

**FIGURE 1:**
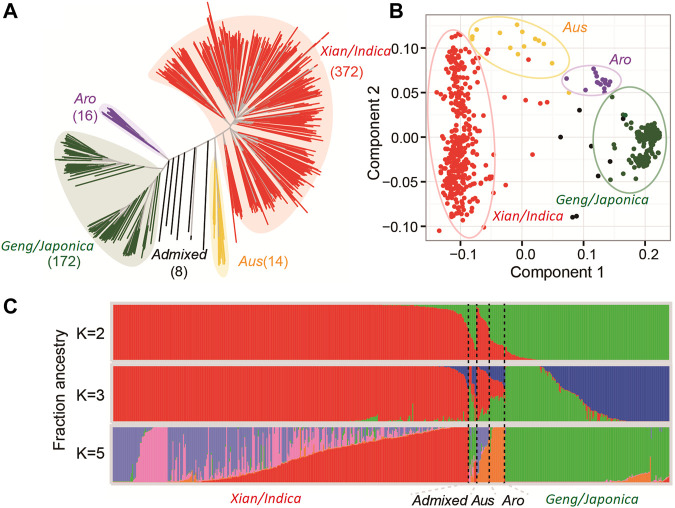
Population structure of germplasm showing NJ tree plot **(A)**, PC **(B)**, and Bayesian clustering **(C)** of germplasm.

The population structure and kinship analyses using the 2,760,730 high-quality SNPs through STRUCTURE indicated that *k* = 2 was most informative to describe the population structure of the 498 accessions used in this study (Figure 1). The huge amounts of variation for all measured AGT and SGT traits (Table 1) were the foundation for our following GWAS analyses, with the whole population consisting of all 498 lines, the *Geng* (*Japonica*) population consisting of 146 accessions (*Geng* + *Bas*), the *Xian* (*Indica*) population consisting of 326 (*Xian* + *Aus*).

### 3.3 Identification of QTLs for AGT and SGT Traits by GWAS

**FIGURE 2:**
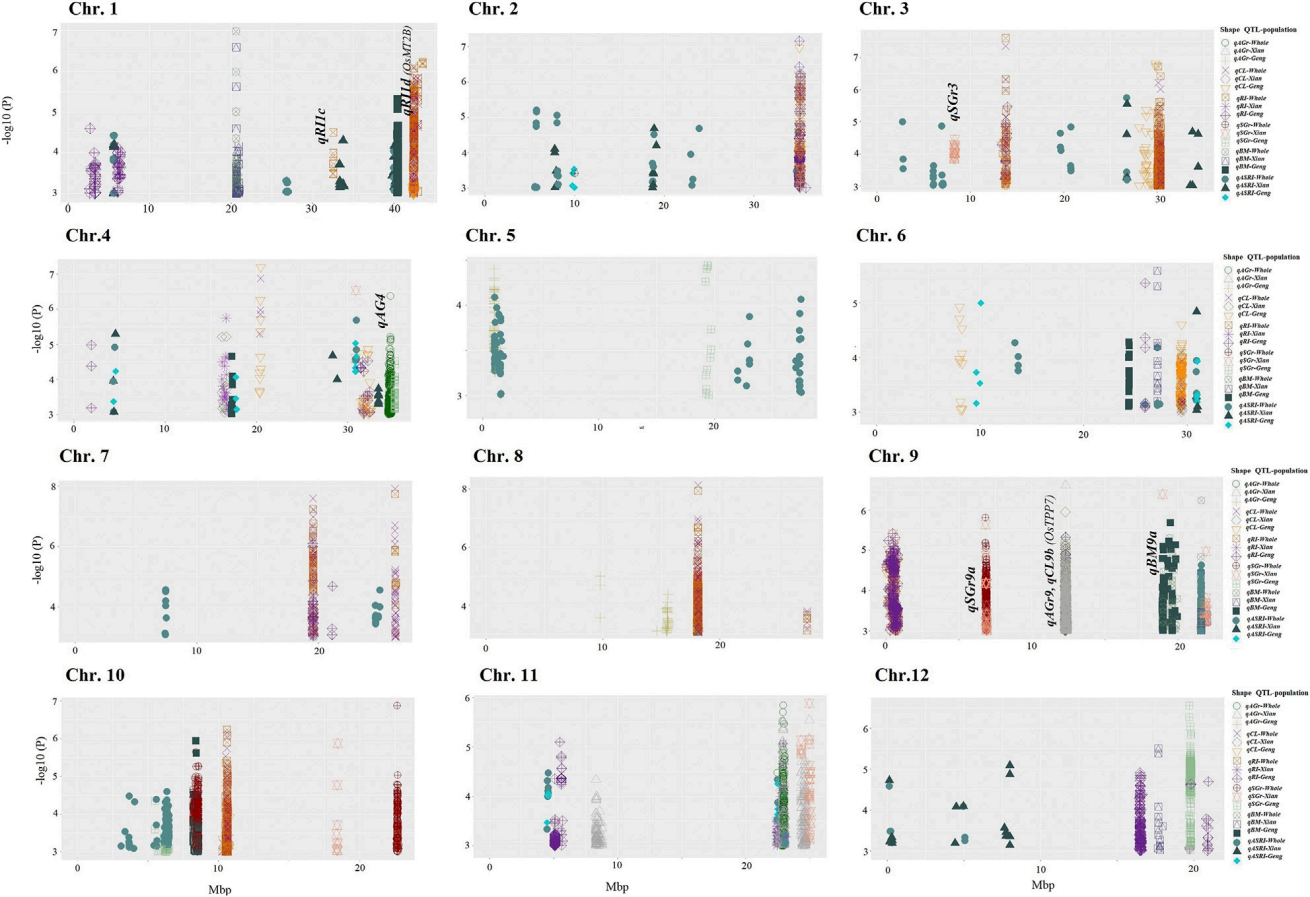
Manhattan plots of AGT and SGT QTLs in the whole genome. Significant SNPs for six traits in *Xian* (*Indica*), *Geng (Japonica*), or whole populations are displayed in different colors and shapes; each shape represents specific QTL identified in *Xian* (*Indica*), *Geng* (*Japonica*), or whole.

Based on a stringent threshold (*p* = 1.0 × 10^−5^ for *Geng* and 1.0 × 10^−6^ for *Xian*), we detected a total 99 QTLs associated with the six traits measured under the salinity and anaerobic germination stresses in different rice populations (Figure 2). For the 3 AGT traits, we identified 54 QTLs. Nine QTLs associated with AGr mapped to chromosomes 2, 4, 5, 8, 9, and 11, four of which (*qAGr2*, *qAGr5*, *qAGr8a*, and *qAGr8b*) were identified only in population *Geng* (*Japonica*), three QTLs (*qAGr9*, *qAGr11a* and *qAGr11c*) only in population *Xian* (*Indica*), and the remaining two (*qAGr4* and *qAGr11b*) in both whole and *Xian* (*Indica*) populations. These QTLs had main effects ranging from 7.1 for *qAGr11a* to 19.5 for *qAGr8b* (Supplementary Table S3). Eighteen QTLs affecting CL were identified and mapped to chromosomes 1, 2, 3, 4, 6, 7, 8, 9, and 10. Eight of these QTLs were detected only in population *Geng* (*Japonica*), *qCL4a* and *qCL9b* were detectable only in population *Xian* (*Indica*), and seven QTLs were detectable only in the whole population, suggesting the allelic differences at these QTLs were primarily reflected between the two subspecies. *qCL4b* was detected in the whole and *Geng* (*Japonica*) populations. These QTLs had effects ranging from 3.3 for *qCL3d* to 10.6 for *qCL9b.* Twenty-seven QTLs affecting anaerobic RI were identified. Of these, 15 were detected only in population *Geng* (*Japonica*), and eight were detectable only in the whole population; *qRI4b*, *qRI9b*, and *qRI11c* only in population *Xian* (*Indica*); and *qRI4c* in both *Geng* (*Japonica*) and whole populations. These RI QTL had effects ranging from 2.3 for *qRI11a* to 16.3 for *qRI12b.* We noted that nine pairs of CL and RI QTLs were mapped to the identical locations with the same peak SNPs (Supplementary Table S3). Each of these cases should be considered the same QTL affecting both CL and RI because of the high positive correlation between CL and RI.

Twenty-one QTLs were identified for the SGT traits. Nine QTLs associated with BM were identified and mapped to chromosomes 1, 4, 6, 9, 10, and 12. These included *qBM1b*, *qBM4*, *qBM6a*, *qBM9b*, and *qBM10* detectable only in population *Geng* (*Japonica*), *qBM6b* and *qBM12* only in population *Xian* (*Indica*), and *qBM1a* in both whole and *Xian* (*Indica*) populations. These QTLs had effects ranging from 3.2 for *qBM6b* to 9.6 for *qBM9a.* Twelve QTLs affecting salt GR (SGr) were identified and mapped to chromosomes 3, 4, 5, 9, 10, 11, and 12. These included *qSGr4b*, *qSGr5*, *qSGr10a*, and *qSGr12* in population *Geng* (*Japonica*); *qSGr3*, *qSGr4a*, *qSGr9b*, *qSGr10c*, and *qSGr11* in population *Xian* (*Indica*); *qSGr10b* and *qSGr10d* in the whole population; and *qSGr9a* in the *Xian* (*Indica*) and whole populations. These QTLs had effects ranging from 4.7 for *qSGr4a* to 20.2 for *qSGr4b* (Supplementary Table S3).

In addition, we identified 42 QTLs associated with ASRI and mapped to all 12 rice chromosomes. Twenty of these QTLs were detected in the whole population; seven QTLs (*qASRI1a*, *qASRI1d*, *qASRI2e*, *qASRI2g*, *qASRI3e*, *qASRI12a*, and *qASRI12b*) in the *Xian* (*Indica*) and whole populations; *qASRI4a* and *qASRI6d* were in all three populations; seven QTLs (*qASRI1c*, *qASRI1e*, *qASRI2b*, *qASRI3f*, *qASRI4c*, *qASRI4e*, and *qASRI12c*) detected only in population *Xian* (*Indica*); *qASRI2d*, *qASRI4b*, and *qASRI6a* in population *Geng* (*Japonica*); and *qASRI4d*, *qASRI11a*, and *qASRI11b* in the *Geng* (*Japonica*) and whole populations. These QTLs had effects ranging from 2.1 ASRI for *qASRI5b* to 11.3 ASRI for *qASRI3e* (Figure 2; Supplementary Table S2).

### 3.4 GO Enrichment Analysis

In total, 317 genes with significant SNP were used for GO enrichment analysis, and significant GO terms for embryonic development, nitrogen compound metabolism, nucleic acid metabolism, carbohydrate metabolic process, response to stress, and response to stimulus were identified. Four GO terms (embryonic development, carbohydrate metabolic process, response to stress, and response to stimulus) highly associated with abiotic stress tolerance were targeted for further analysis, and 22 genes were found common for the target GO terms. The results explained that the genes with significant SNPs are associated with abiotic stress tolerance trait in rice (Supplementary Figures S4, S5).

### 3.5 Candidate Genes for Important QTLs

Based on the GWAS results, we were able to determine seven important QTLs: *qRI1c, qRI1d, qSGr3, qAGr4, qBM9a, qSGr9a*, and *qAGr9* (*qCL9b* and *qRI9b*), based on their large phenotypic effects and LOD peaks of multiple highly significant SNPs mapped to a small region of ∼1 Mb or to a fine mapped QTL/cloned gene of functional relevance. Based on the criteria, four QTLs, *qRI1c, qRI1d, qAGr4*, and *qAGr9* (*qCL9b* and *qRI9b*), with large effects on AGT, appeared to be more important, and candidate genes were identified in those regions. *qAGr4* was mapped in a confidence interval of 230 kb (34.60–34.83 Mb) on chromosome 4 with 142 SNPs in the 32 genes, which resulted in identification of six candidate genes, *Os04g0677700*, *Os04g0678300*, *Os04g0678700*, *Os04g0679050*, *Os04g0681600*, and *Os04g0682100* (Table 2). *Os04g0679050* encoding an H0801D08.10 protein and has 3 major haplotypes consisted of nonsynonymous SNPs. Hap1 presented in only 3 *Xian* (*Indica*) and 11 *Geng* (*Japonica*) accessions and had a mean AGr of 45.4%, significantly higher than the predominant Hap2 (mean AGr = 31.3%) and Hap3 (mean AGr = 11.3%) (Supplementary Figure S3). Similarly, *Os04g0682100* encodes a C2 calcium/lipid-binding protein and has only two major haplotypes in the whole population. Hap1 was predominant in population *Geng* (*Japonica*) and associated with a mean AGr of 57.3%, significantly higher than Hap2, which had a mean AGr of 21.8% and was predominant in population *Xian* (*Indica*) (Supplementary Figure S3). We observed four major haplotypes at *Os04g0681600*, which encodes a DUF580 family protein of unknown function. Hap1 and Hap2 were present in population *Geng* (*Japonica*) and had mean AGr of 54.4% and 44.2%, significantly higher than Hap3 and Hap4, which were present primarily in population *Xian* (*Indica*) (Supplementary Figure S3; Table 3). These results suggested *Os04g0679050*, *Os04g0681600*, and *Os04g0682100* were most likely candidates for *qAGr4*. *qAGr9* (*qCL9b* and *qRI9b*) mapped to an interval of 240 kb (12.20–12.44 Mb) on chromosome 9, which harbors a cloned QTL gene, *OsTPP7* (*LOC_Os09g20390*), encoding the trehalose-6-phosphate phosphatase involved in trehalose-6-phosphate metabolism and enhancing rice AGT by driving growth kinetics of the germinating embryo and elongating coleoptile under anaerobic conditions (Kretzschmar et al., 2015). The haplotype analyses suggest seven candidate genes, *Os09g0369050*, *Os09g0369250*, *Os09g0369400*, *Os09g0369500*, *Os09g0370500*, *Os09g0371000*, and *Os09g0372800*. Of them, *Os09g0369400* encodes a protein similar to trehalose-6-phosphate phosphatase 7 (TPP7) and has only three major haplotypes in population *Xian* (*Indica*) with Hap1 associated with significantly higher AGr and longer CL than Hap2 (Supplementary Figure S3; Table 3).

**TABLE 2:** List of 25 candidate genes for seven important QTLs identified at under salinity and anaerobic stresses.

Sr. No	QTLs	Loci	Annotation
1	*qRI1c*	*Os01g0772500*	Glycosyl transferase
2	*qRI1d*	*Os01g0974200*	RicMT (metallothionein-like protein), conserved hypothetical protein (*MT2B*)
3	*qRI1d*	*Os01g0976100*	ABC transporter-like domain–containing protein
4	*qSGr3*	*Os03g0230300*	Regulation of stomatal closure, abiotic stress response
5	*qSGr3*	*Os03g0231700*	Squalene monooxygenase, putative, expressed
6	*qSGr3*	*Os03g0231800*	Similar to squalene monooxygenase
7	*qSGr3*	*Os03g0233000*	Protein of unknown function DUF607 family protein
8	*qAGr4*	*Os04g0677700*	Similar to H0402C08.11 protein
9	*qAGr4*	*Os04g0678300*	WD-40 repeat family protein, putative, expressed
10	*qAGr4*	*Os04g0678700*	Similar to protochlorophyllide reductase
11	*qAGr4*	*Os04g0679050*	Similar to H0801D08.10 protein
12	*qAGr4*	*Os04g0681600*	Protein of unknown function DUF580 family protein
13	*qAGr4*	*Os04g0682100*	C2 calcium/lipid-binding region, CaLB domain–containing protein
14	*qAGr9, qCL9b*	*Os09g0369050*	Similar to DRE-binding factor 2
15	*qAGr9, qCL9b*	*Os09g0369250*	Expressed protein
16	*qAGr9, qCL9b*	*Os09g0369400*	Similar to trehalose-6-phosphate phosphatase 7 (*TPP7*)
17	*qAGr9, qCL9b*	*Os09g0369500*	Endosperm-specific gene 127
18	*qCL9b*	*Os09g0370500*	VQ domain–containing protein
19	*qAGr9*	*Os09g0371000*	Major facilitator superfamily protein
20	*qAGr9*	*Os09g0372800*	Serine/threonine protein kinase domain–containing protein
21	*qBM9a*	*Os09g0490200*	Similar to ethylene signal transcription factor
22	*qBM9a*	*Os09g0490400*	β-Glucosidase 29
23	*qBM9a*	*Os09g0491740*	Auxin efflux carrier domain–containing protein
24	*qBM9a*	*Os09g0493700*	Similar to CUC2
25	*qSGr9a*	*Os09g0306400*	bZIP transcription factor, drought and salt tolerance

**TABLE 3:** Haplotype analysis of the candidate genes for important QTL regions.

QTLs	Genes	Hap	SNPs	*Xian* (*Indica*)	*Geng* (*Japonica*)	Other	Total	Mean	SD
*qAGr4*	*Os04g0679050*	Hap-1	CC	3	11	0	14	45.4	8.0
		Hap-2	CT	269	109	24	402	31.3	9.1
		Hap-3	TC	30	0	0	30	11.3	5.2
	*Os04g0681600*	Hap-1	GCA	0	12	5	17	54.4	9.9
		Hap-2	ACG	0	12	0	12	44.2	9.3
		Hap-3	GCG	14	0	4	18	28.1	7.6
		Hap-4	GTG	293	56	5	354	23.4	6.5
	*Os04g0682100*	Hap-1	T	3	90	9	102	57.3	9.1
		Hap-2	C	291	5	12	308	21.8	9.1
*qBM9a*	*Os09g0490200*	Hap-1	AGCT	11	3	0	14	1.5	0.2
		Hap-2	GATC	271	113	22	406	1.1	0.1
	*Os09g0490400*	Hap-1	GTT	286	0	4	290	1.4	0.2
		Hap-2	GGT	5	104	3	112	1.1	0.2
		Hap-3	AGC	2	0	15	17	0.9	0.1
	*Os09g0493700*	Hap-1	TGG	75	0	1	76	1.4	0.2
		Hap-2	TCA	19	0	10	29	1.1	0.2
		Hap-3	GCG	4	29	1	34	0.9	0.1
*qAGr9*	*Os09g0369400*	Hap-1	CA	98	22	14	134	51.4	9.4
		Hap-2	GA	152	4	0	156	27.3	10.8
		Hap-3	GC	34	2	0	36	10.3	5.3
	*Os09g0369500*	Hap-1	AAA	21	88	12	121	55.6	10.09
		Hap-2	CGC	143	6	3	152	30.6	9.0
		Hap-3	CGA	20	2	0	22	10.1	4.4
*qCL9b*	*Os09g0369400*	Hap-1	CA	100	20	0	120	3.54	0.58
		Hap-2	GA	146	4	0	150	2.85	10.8
		Hap-3	GC	36	6	0	42	2.16	0.20
*qSGr3*	*Os03g0231800*	Hap-1	AGC	0	73	2	75	44.4	7.1
		Hap-2	TGC	12	1	16	29	19.0	8.8
		Hap-3	TTT	173	5	20	198	15.8	8.6
		Hap-4	TGT	14	0	0	14	10.8	8.7

**FIGURE 3:**
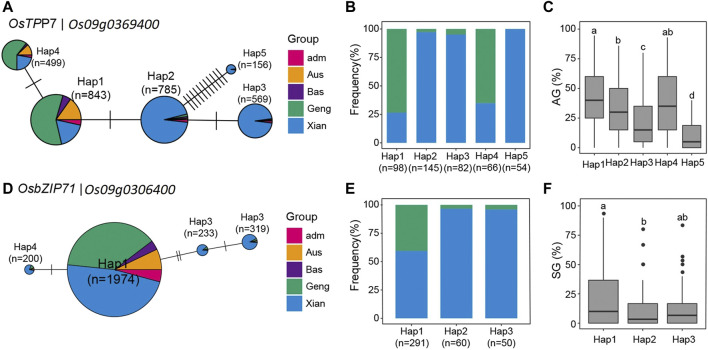
**(A)** for OsTPP7 and **(D)** for OsbZIP71; Frequencies of **(B)**, 5 haplotypes(Hap) of OsTPP7 and **(E)**, 3 haplotypes(Hap) of OsbZIP71 in subgroups of 3RGP; The distribution of AG for the **(C)**, 5 Haps of OsTPP7 and the distribution of SG the **(F)**, 3 Haps of OsbZIP71. Different letters above each boxplot indicate significant differences among haplotypes according to Tukey’s honest’s significant difference test (*p* < 0.05).

We discovered five major haplotypes (with frequency >150) constructed from SNPs within the CDS regions of this gene in the 3,010 rice accessions (Figure 3A). According to their frequencies in the five major rice populations, Hap1 and its derived one, Hap4, were the predominant alleles in population *Geng* (*Japonica*) and associated with high AGr in the tested populations, whereas Hap2 was the predominant allele in population *Xian* (*Indica*) and associated with lower AGr. However, two less frequent alleles, Hap3 and Hap5, which were present almost only in population *Xian* (*Indica*), were associated with greatly reduced AGr. Figure 3D explains that *Os09g0306400* has four haplotypes in the 3,010 rice accessions, and according to the frequencies, haplotypes were the predominant alleles in population *Xian* (*Indica*), and this allele is associated with high SGr in the tested populations. On chromosome 1, three candidate genes, *Os01g0772500*, *Os01g0974200*, and *Os01g0976100*, were identified in two important QTLs (*qRI1c* and *qRI1d*). *Os01g0772500* encodes glycosyl transferase, *Os01g0974200* encodes RicMT (metallothionein-like protein), conserved hypothetical protein (*MT2B*), and *Os01g0976100* encodes protein contains domain for ABC transporter.

Three SGT important QTLs, *qSGr3*, *qBM9a*, and *qSGr9a*, were analyzed for candidate gene identification. *qSGr3* mapped to chromosome 3 in the confidence interval of 360 kb (6.83–7.19 Mb) where we detected 158 SNPs in the 41 genes with LOD peaks in genes *Os03g0230300* (regulation of stomatal closure and abiotic stress response), *Os03g0231700* (the squalene monooxygenase), *Os03g0231800* (similar to squalene monooxygenase), and *Os03g0233000* (a DUF607 family protein of unknown function) (Figure 3), indicating that they were the most likely candidate genes for *qSGr3*. *Os03g0231800* is predicted to encode a squalene monooxygenase and has four major haplotypes in the mapping panel populations. Hap1 was associated with significantly higher SGr than the other three haplotypes based on ANOVA (Supplementary Figure S3). At the other three candidate genes, no significant differences were detected among their haplotypes, suggesting *Os03g0231800* can be candidate gene for *qSGr3*.

*qBM9a* was mapped to a confidence interval of 260 kb (18.88–19.14) on chromosome 4–containing 118 SNPs with 3 LOD peaks in 21 genes, which led us to identify four most likely candidate genes, *Os09g0490200* (similar to ethylene signal transcription factor), *Os09g0490400* (β-glucosidase 29), *Os09g0491740* (an auxin efflux carrier domain–containing protein), and *Os09g0493700* (similar to CUC2). *Os09g0490400* encodes the β-glucosidase 29 and has three major haplotypes in the whole population. Hap1 was associated with the highest BM, followed by Hap2 and Hap3 with the lowest BM (Table 3; Supplementary Figure S3). Predicted to be an ethylene signal transcription factor gene, *Os09g0490200* has two major haplotypes, and Hap1 was associated with significantly higher BM (Supplementary Figure S3). *Os09g0493700* is predicted to be a CUC2 gene with three major haplotypes, and Hap1 was associated with the highest BM, followed by Hap2, and Hap3 had the lowest BM (Supplementary Figure S3).

## 4 Discussion

DSR has increased rapidly recently in different Asian countries because of its being labor-saving and cost-effective. However, developing high-yielding rice cultivars suitable for direct seeding must have good AGT and SGT in order to achieve high and sustainable yields under salinity and anaerobic stresses at the germination stage. In this study, we have shown that there is tremendous genetic variation for traits contributing to AGT and SGT in the primary gene pool of rice and identified large numbers of QTLs affecting the AGT and SGT traits in rice. While providing a clear picture regarding the overall level and pattern of this useful genetic diversity and materials, our results shed some light on the genetic basis of SGT and AGT in rice and suggest more efficient strategies how to exploit this valuable genetic variation in future development of new and high-yielding DSR varieties. In this study, huge amounts of phenotypic variation were observed among accessions for the measured AGT and SGT traits, primarily within the subspecific populations. On average, *Geng* (*Japonica*) accessions showed significantly higher AGT than *Xian* (*Indica*) accessions, which was also reflected by more *Geng* (*Japonica*) accessions showing high level of AGT. The subspecific differences in SGT traits were less pronounced. Thus, future improvement of both AGT and SGT should focus on exploitation of within-subspecies variation. In this respect, the identification of 33 AGT accessions and 14 SGT accessions of diverse origins (Table 2) provided excellent materials for future genetic/molecular dissection and improvement of AGT and SGT.

With the threshold of −log10 (*p *< 10^−5^) for two or more SNPs in 200 kb by GWAS, we were able to identify large numbers of QTLs significantly associated with the AGT and SGT traits and ASGT, indicating rice adaptation to AG, SG, and ASG stresses is genetically complex. Clearly, most of the identified QTLs had relatively small effects on the AG, SGT traits, and ASGT. We noted that most (87.1%) of the identified QTLs were detected in a single population, including 39 QTLs detectable only in population *Geng* (*Japonica*), 22 QTLs in population *Xian* (*Indica*), and 39 QTLs detected only in the whole population, whereas 5 (*qCL4b*, *qRI7a*, *qASRI4d*, *qASRI11a*, and *qASRI11b*) and 10 QTLs (*qAGr11b*, *qBM1a*, *qSGr9a*, *qASRI1a*, *qASRI1d*, *qASRI2e*, *qASRI2g*, *qASRI3e*, *qASRI12a*, and *qASRI12b*) were detected in whole in parallel with *Geng* (*Japonica*) and *Xian* (*Indica*) populations, respectively, and 2 QTLs (*qASRI4a* and *qASRI6d*) were detected in *Geng*, *Xian* (*Indica*) and whole populations. While providing strong evidence for the impact of population structure of the tested accessions on effectiveness of GWAS, this result has important implications for their potential application to improving rice AGT, SGT, and ASGT in future breeding programs, for example, for those QTLs identified in either population *Xian* (*Indica*) or *Geng* (*Japonica*).

Three QTL (*qCL9b*, *qRI9b*, and *qAGr9*) associated with AGT were identified in the same region on chromosome 9 (12.20–12.44 Mb); this region was previously cloned and reported as major anaerobic stress tolerance gene *OsTPP7* (Kretzschmar et al., 2015). Two QTL *qBM9a* and *qBM9b* associated with salinity stress were identified in the same region on chromosome 9 (18.87–20.00 Mb); this region was previously cloned from *Xian* (*Indica*) rice variety 9,311 and reported as *OsEATB*, a major gene involved in the reduction of plant height that is a major factor to increase plant yield (Qi et al., 2011). One QTL (*qRI1c*) was identified on chromosome 1 (32.60–33.03 Mb); this region is adjacent to the previously cloned gene *OsKAT1*, which was reported as a rice shaker potassium channel that confers tolerance to salinity stress in rice (Obata et al., 2007). One QTL (*qSGr9a*) was identified on chromosome 9 (6.82–7.05 Mb); this region is adjacent to previously cloned gene *OsbZIP71*, another rice shaker potassium channel conferring salinity tolerance in rice (Liu et al., 2013). Two QTLs (*qRI12b and qSGr12*) were identified in the same region on chromosome 12 (19.68–19.84) associated with SGr and AGr length; this region is adjacent to previously cloned gene *OsATG10b* (Shin et al., 2009), which plays an important role in the survival of rice cells against oxidative stresses. One QTL (*qRI1d*) was identified on chromosome 1 associated with root length; this region was previously reported for salt tolerance as *OrbHLH001*, and overexpression of *OrbHLH001* from Dongxiang wild rice (*Oryza rufipogon*) conferred salt tolerance in rice plants (Chen et al., 2013). One QTL (*qSGr3*) was identified on chromosome 3 associated with salinity germination; this region was reported for drought and salt tolerance as dsm3 (Du et al., 2011). One region of chromosome 10 (8.29–8.58 Mb) had three QTLs (*qSGr10b* and *qBM10*) associated with two traits measured under salinity stress; these traits have positive correlations with each other. Association of this region with three different traits predicts that this region can produce salinity tolerance. A major advantage in our study was the use of high-density genotypic data to identify QTL through GWAS, allowing us to narrow down the QTL region to <200 kb, which we further investigated to identify significant SNPs >−log (*p*) > 3 in the CDS to mark candidate genes. In total, 24 candidate genes were identified for 7 QTL short-listed as important. One candidate gene, *Os01g0772500* for *qRI1*, encodes glycosyl transferase, and in rice, this protein has been characterized as leaf senescence protein and associated with photosynthetic rate, stomatal conductance, and transpiration rate (Wang M. et al., 2018).

Two candidate genes, *Os01g0974200* and *Os01g0976100*, were identified for *qRI1d*. *Os01g0974200* encodes protein RicMT (metallothionein-like protein), and metallothionein-producing genes are involved in multiple types of abiotic stress tolerance (Kumar et al., 2012). Identification of this locus in our study can be cloned to find genes for abiotic stress tolerance. While *Os01g0976100* encodes a protein-containing ABC transporter-like domains, these proteins are involved in plant developmental processes and transporting various compounds/elements across cell membranes (Hwang et al., 2016). Four candidate genes, *Os03g0230300*, *Os03g0231700*, *Os03g0231800*, and *Os03g0233000*, were identified for *qGS3*. *Os03g0230300* encodes a protein involved in the regulation of stomatal closure and the abiotic stress response (You and Chan, 2015). *Os03g0231700* encodes squalene monooxygenase, and Manavalan et al. (2011) used RNAi-mediated disruption of squalene synthase and found drought tolerance and improvement in rice yield. *Os03g0231800* expressed a putative protein, and *Os03g0233000* encodes a protein of unknown function in the DUF607 family. DUF domain proteins are reported for drought tolerance in rice (Cui et al., 2016). Six candidate genes, *Os04g0677700*, *Os04g0678300*, *Os04g0678700*, *Os04g0679050*, *Os04g0681600*, and *Os04g0682100*, were identified for *qAGr4*. *Os04g0677700* and *Os04g0679050* expressed putative proteins similar to *H0402C08.11* and *H0801D08* protein. *Os04g0678300* expressed a WD-40 family protein; these proteins are associated with plant tolerance to abiotic stresses (Kong et al., 2015). *Os04g0678700* expressed protochlorophyllide reductase. Protochlorophyllide is a precursor of chlorophyll, which is the most important component of photosynthesis and anabolic processes (Dalal and Tripathy, 2012). *Os04g0681600* expressed a DUF580 domain protein. *Os04g0682100* expressed a CaLB domain protein, which is a novel repressor of abiotic stress responses (de Silva et al., 2011). Five candidate genes, *Os09g0369050*, *Os09g0369250*, *Os09g0369400*, *Os09g0369500*, and *Os09g0370500*, were identified for *qCL9b*, and six candidate genes, *Os09g0369050*, *Os09g0369250*, *Os09g0369400*, *Os09g0369500*, *Os09g0371000*, and *Os09g0372800*, for *qAGr9*. Four candidate genes (*Os09g0369050*, *Os09g0369250*, *Os09g0369400*, and *Os09g0369500*) were significant for both QTL *qAGr9* and *qCL9b*. *Os09g0369050* expressed a protein similar to DRE-binding factor 2 protein. DRE elements are present in the promoter regions of various gene involved in abiotic stress tolerance. *Os09g0369250* expressed a putative protein of unknown function, and *Os09g0369400* expressed a protein similar to trehalose-6-phosphate, phosphatase 7, as *osTPP7*, a cloned gene for anaerobic stress tolerance at germination in rice (Kretzschmar et al., 2015), and it participates in starch mobilization to promote embryo germination and coleoptile elongation (Hsu and Tung, 2015). *Os09g0369500* expressed a conserved protein known as endosperm-specific gene (*OsEnS*). The endosperm is a critical factor for seed growth, and *OsEnS* gene was identified on chromosome 9 in rice (Nie et al., 2013). *Os09g0370500* expressed a VQ domain protein; VQ domain proteins are involved in abiotic stress responses and developmental processes (Jing and Lin, 2015). *Os09g0371000* expressed a major facilitator superfamily protein, and these proteins have multiple roles in auxin transport and drought stress tolerance in *Arabidopsis* (Remy et al., 2013). *Os09g0372800* expressed a serine/threonine protein kinase domain–containing protein, and this protein causes resistance to rice stripe disease (Lee and Kim, 2015). Four candidate genes, *Os09g0490200*, *Os09g0490400*, *Os09g0491740*, and *Os09g0493700*, were identified for *qBM9a*. *Os09g0490200* encodes ethylene signal transcription factor, and it is reported that ethylene signaling-related genes respond to dehydration stresses (Ren et al., 2017). *Os09g0490400* expressed β-glucosidase; these proteins are involved in abiotic stresses through the accumulation of antioxidant flavanols (Baba et al., 2017). *Os09g0491740* expressed auxin efflux carrier domain protein, and auxin has been reported as a key growth regulator that is involved in abiotic stress responses (Sharma et al., 2015). *Os09g0493700* expressed a protein similar to CUC2; CUC is associated with drought and salt tolerance in rice.

Identification of the candidate gene based on its relevance in the mechanism to the trait of interest leads us to identify trait-controlling genes. Identification of new and previously reported QTL/candidate genes in this study demonstrated the advantages of GWAS using high genetic diversity and higher-resolution mapping to identify candidate genes. However, few confines were found for GWAS approach, as we had a limited ability to detect rare QTL/alleles because minor alleles were removed, and this method cannot detect epistasis. A further selection of nonsynonymous SNPs in the coding regions of the gene for haplotype analyses cannot cover the trait variation caused by SNP/mutation in the promoter or noncoding regions of the gene. Moreover, use of a single reference genome (presence or absence of gene) can cause errors in GWAS for the accuracy of QTL and candidate genes.

## 5 Conclusion

In GWAS, using high genetic diversity is a powerful tool for the identification of QTL candidate genes and haplotypes. In this study, a total of 54 and 21 QTLs were identified related to anaerobic and salt tolerance at the germination stage. Different genomic regions of *Xian* (*Indica*) and *Geng* (*Japonica*) are involved in AGT and SGT trait, which suggest specific QTL for the subgroup. In total, 25 candidate genes were identified, several of these in the genomic regions reported or cloned for anaerobic and salt tolerance. It was also found the *Geng* (*Japonica*) accession having more tolerance to anaerobic stress as compared to the *Xian* (*Indica*). The identified anaerobic and salt tolerant accessions that have high breeding values can be used in future rice breeding for anaerobic and salinity tolerance at germination stage. Identification of tolerant accessions and the QTLs/genes in this study supports that results are useful for the ongoing and future rice breeding programs.

## Data Availability

The original contributions presented in the study are included in the article/Supplementary Material, further inquiries can be directed to the corresponding authors.

## Supplementary Material

Supplementary Figure 1 Linkage disequilibrium (LD) decay and comparison. The genome‐wide LD decay comparison between Geng, Xian, and whole populations.

Supplementary Figure 2 Correlations between the measured traits; intensity of blue and red colors indicates positive and negative correlations, respectively.

Supplementary Figure 3 Manhattan plot of important QTL and haplotype analysis of candidate genes. These genes are related to corresponding QTL including analysis of candidate genes done by using SAS general linear model approach (**(A)**: qBM9a, **(B)**: qSGr3, **(C)**: qAGr4, **(D)**: qCL9b, **(E)**: qAGr9). Each point was a gene in the 200-kb region of the peak SNP of QTL. Dot line showed the threshold to determine candidate genes. The suggested significance of ANOVA at *p* < 0.01. The letters on histogram (a and b) show multiple comparisons result at the significant level of 0.01. The value on top of histogram was the number ofindividuals for each haplotype, and histogram length shows the mean value of these individuals for each haplotype.

Supplementary Figure 4 Gene Ontology (GO) terms that enrich significant genes. The color indicates the degree of enrichment from red (highly enriched and significant) to white (not enriched).

Supplementary Figure 5 Gene Ontology (GO) comparison of GO terms for biological processes is significantly associated with the traits of AGT, SGT and ASGT. The term overlap shows the four ways of constructing graphs.
