# Supplementary figures and images for: Identification of Candidate Genes for Salinity and Anaerobic Tolerance at the Germination Stage in Rice by Genome-Wide Association Analyses
Source: Front Genet. 2022 Feb 23;13:822516. doi: 10.3389/fgene.2022.822516 (PMC8905349; doi:10.3389/fgene.2022.822516)

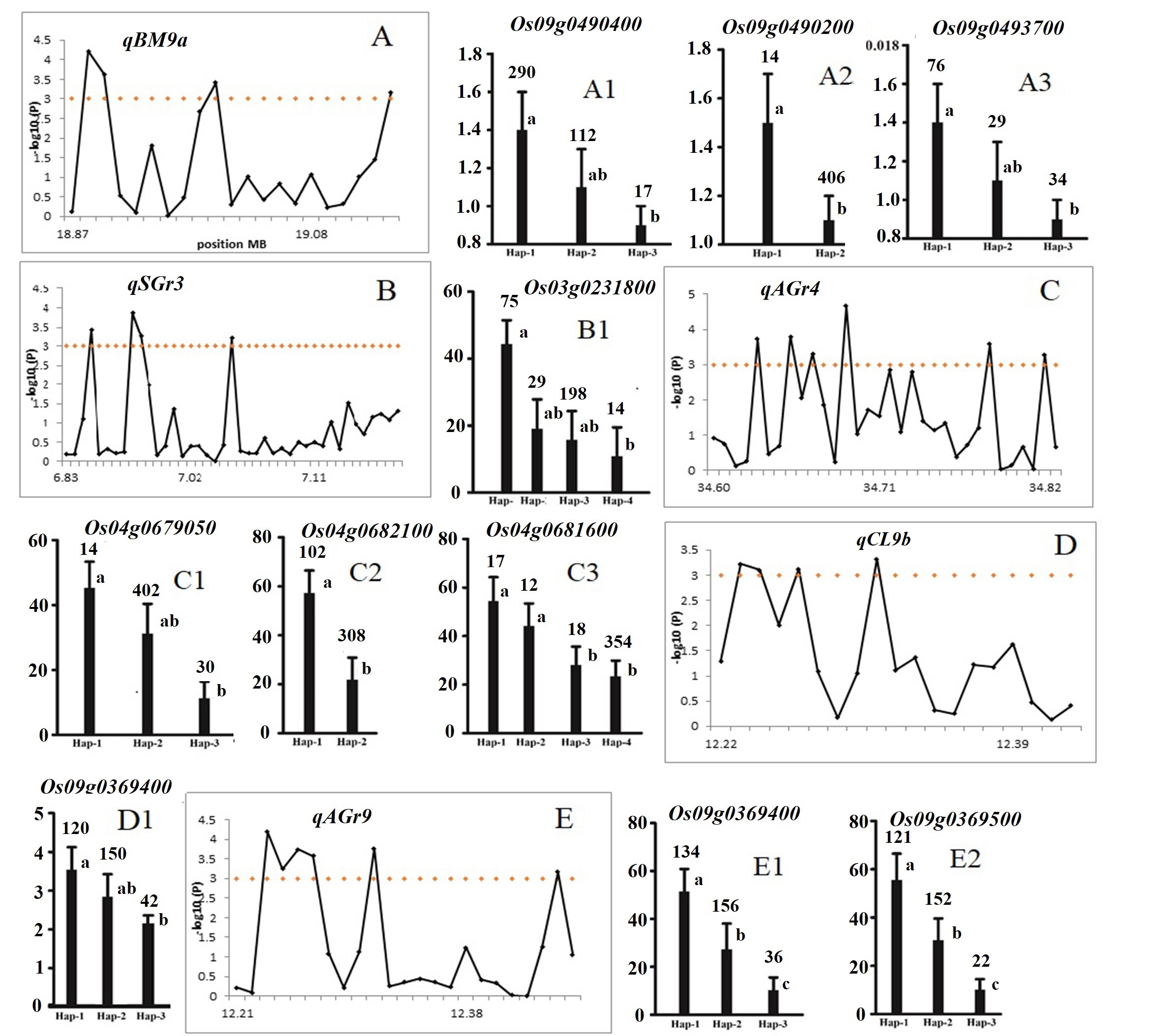

Supplement: Supplementary file 1 [file Image3.JPEG]

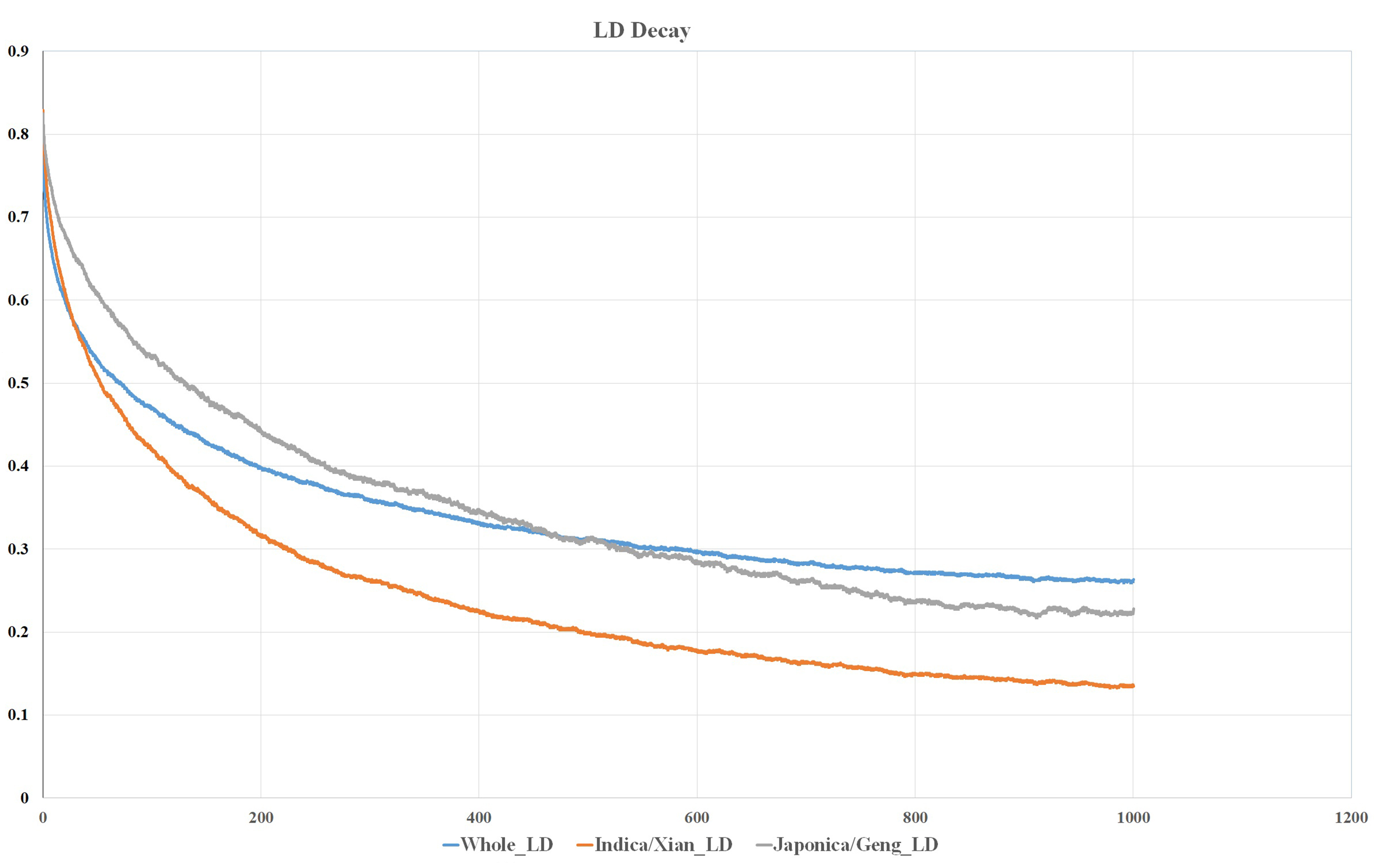

Supplement: Supplementary file 2 [file Image1.JPEG]

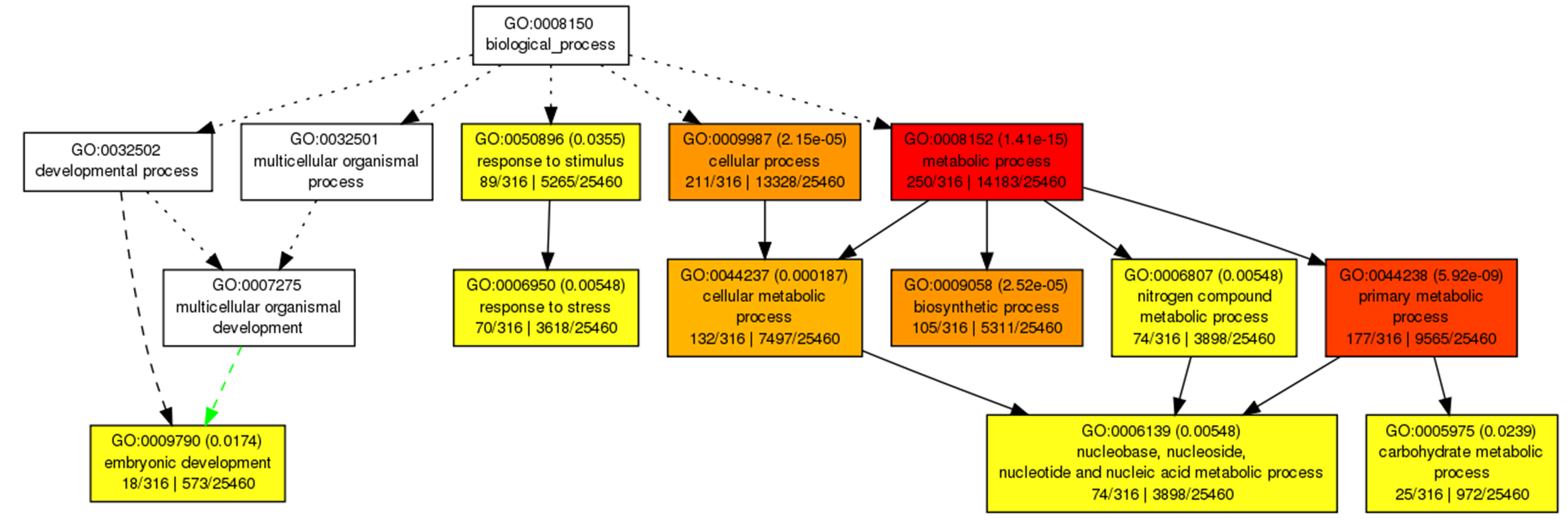

Supplement: Supplementary file 3 [file Image4.JPEG]

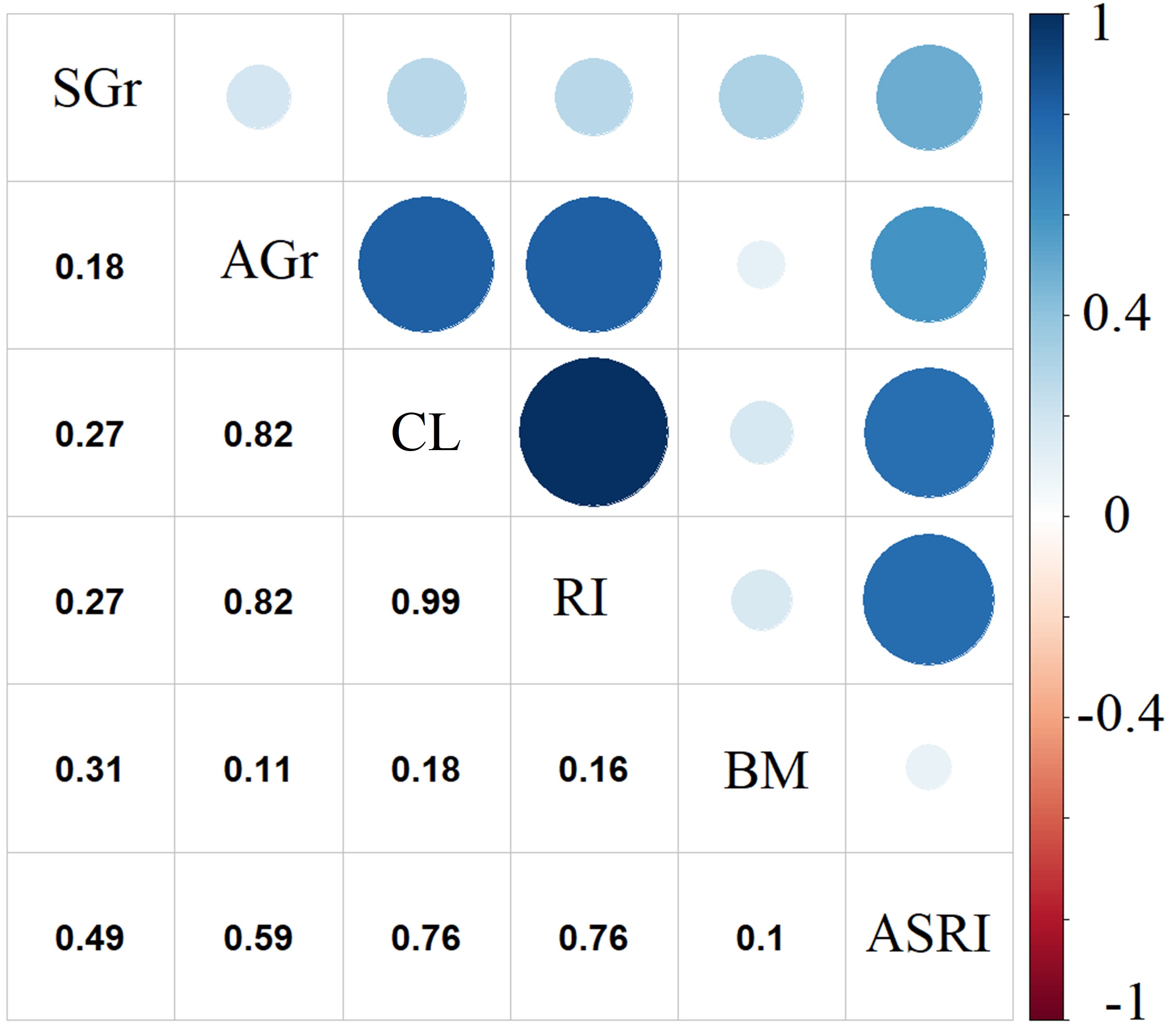

Supplement: Supplementary file 4 [file Image2.JPEG]

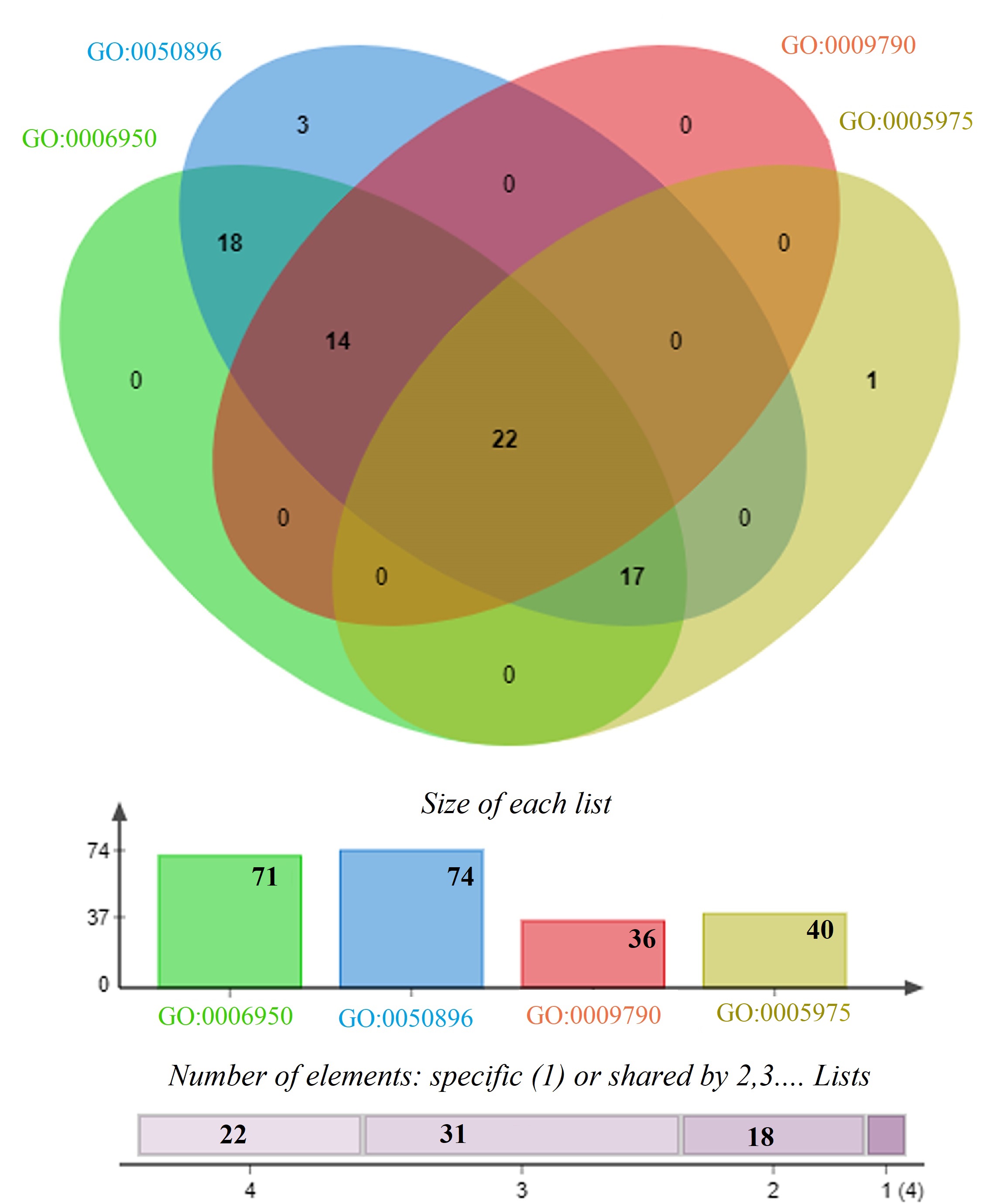

Supplement: Supplementary file 5 [file Image5.JPEG]
