# Supplementary material for: Identification of Candidate Genes for Salinity and Anaerobic Tolerance at the Germination Stage in Rice by Genome-Wide Association Analyses
Source: Front Genet. 2022 Feb 23;13:822516. doi: 10.3389/fgene.2022.822516 (PMC8905349; doi:10.3389/fgene.2022.822516)
Supplement: Supplementary file 6 [file DataSheet1.docx]

Table S1. ANOVA for the measured traits at the germination stage under salinity and anaerobic stresses

| **Trait** | **Source of variation** | **df** | **SS** | **MS** | **F** | **P- value** | **R^2^_G_ (%)** |
| --- | --- | --- | --- | --- | --- | --- | --- |
| AGr | Genotype | 497 | 503126.47 | 1012.33 | 34.31 | 0.00 | \|  \| \| --- \| \|  \| \|  \| \| 32.0 \| \|  \| \|  \| \| 97.5 \| \|  \| \|  \| \| 97.4 \| \|  \| \|  \| \| 2.5 \| \|  \| \|  \| \| 11.6 \| \|  \| \|  \| \| 90.9 \| \|  \| \|  \| \| 97.1 \|   97.2 |
|  | Rep | 1 | 42.19 | 42.19 | 1.43 | 0.23 | 97.2 |
|  | Error | 497 | 14662.31 | 29.50 |  |  |  |
| CL | Genotype | 497 | 482.41 | 0.97 | 47.03 | 0.00 | \|  \| \| --- \| \|  \| \|  \| \| 97.5 \| \|  \| \|  \| \| 97.4 \| \|  \| \|  \| \| 2.5 \|   97.8 |
|  | Rep | 1 | 0.033 | 0.033 | 0.016 | 0.899 | 32.0 |
|  | Error | 497 | 10.80 | 2.06 |  |  |  |
| RI | Genotype | 497 | 532572.89 | 1071.58 | 38.38 | 0.00 |  |
|  | Rep | 1 | 95.39 | 95.39 | 3.42 | 0.07 | 97.5 |
|  | Error | 497 | 13874.93 | 27.92 |  |  |  |
| SGr | Genotype | 497 | 293856.74 | 591.26 | 37.22 | 0.00 |  |
|  | Rep | 1 | 101.16 | 101.16 | 6.37 | 0.01 | 97.4 |
|  | Error | 497 | 7895.00 | 15.89 |  |  |  |
| BM | Genotype | 497 | 0.858 | 0.002 | 9.13 | 0.00 |  |
|  | Rep | 1 | 0.0006 | 0.0007 | 3.44 | 0.064 | 90.9 |
|  | Error | 497 | 0.093 | 0.0002 |  |  |  |
| ASRI | Genotype | 198 | 11937.79 | 60.29 | 33.37 | 0.00 |  |
|  | Rep | 1 | 64.26 | 64.26 | 35.57 | 0.00 | 97.1 |
|  | Error | 198 | 357.63 | 1.80 |  |  |  |

Table S2. List of the accessions tolerant for AGT, SGT and ASGT

| **Trait** | **Mean** | **3K_DNA_IRIS_ID** | **Country** | **Group** |
| --- | --- | --- | --- | --- |
| AGT | 82.5 | IRIS_313-11297 | India | admix |
| AGT | 82.5 | IRIS_313-11142 | Myanmar | XI-3 |
| AGT | 82.5 | IRIS_313-10564 | Japan | GJ-tmp |
| AGT | 82.5 | IRIS_313-11571 | China | GJ-tmp |
| AGT | 82.5 | IRIS_313-10558 | China | GJ-tmp |
| AGT | 82.5 | IRIS_313-11654 | China | GJ-tmp |
| AGT | 82.5 | IRIS_313-10959 | Indonesia | GJ-trp |
| AGT | 82.5 | IRIS_313-11698 | Chinese Taipei | cB (Bas) |
| AGT | 82.5 | B086 | China | admix |
| AGT | 82.5 | IRIS_313-9724 | China | GJ-tmp |
| AGT | 81.0 | CX143 | Iran | cB (Bas) |
| AGT | 81.0 | IRIS_313-10710 | Surinam | GJ-trp |
| AGT | 80.0 | IRIS_313-10543 | India | cA (Aus) |
| AGT | 80.0 | B056 | China | GJ-tmp |
| AGT | 80.0 | B199 | China | GJ-tmp |
| AGT | 80.0 | B068 | China | GJ-tmp |
| AGT | 80.0 | IRIS_313-8140 | China | GJ-tmp |
| AGT | 80.0 | IRIS_313-8481 | China | GJ-tmp |
| AGT | 78.5 | IRIS_313-10859 | India | XI-adm |
| AGT | 75.0 | IRIS_313-11408 | India | XI-2 |
| AGT | 75.0 | B084 | China | admix |
| AGT | 79.0 | IRIS_313-11702 | United States of America | GJ-tmp |
| AGT | 82.5 | IRIS_313-11627 | Nepal | cB (Bas) |
| AGT | 76.0 | IRIS_313-10257 | Nicaragua | XI-adm |
| AGT | 75.0 | IRIS_313-11987 | Philippines | XI-adm |
| AGT | 77.5 | IRIS_313-11885 | China | XI-1A |
| AGT | 76.0 | B183 | Japan | GJ-tmp |
| AGT | 77.5 | IRIS_313-11584 | China | GJ-tmp |
| AGT | 77.5 | CX315 | North_Korea | GJ-tmp |
| AGT | 75.0 | IRIS_313-11651 | China | GJ-tmp |
| AGT | 75.0 | IRIS_313-11983 | Burkina Faso | XI-2 |
| AGT | 75.0 | IRIS_313-11582 | China | GJ-tmp |
| AGT | 75.0 | B241 | China | GJ-sbtrp |
| SGT | 82.5 | IRIS_313-11834 | Thailand | GJ-sbtrp |
| SGT | 76.7 | IRIS_313-10710 | Surinam | GJ-trp |
| SGT | 82.5 | IRIS_313-7699 | Philippines | XI-adm |
| SGT | 76.7 | IRIS_313-7912 | Cote d'Ivoire | GJ-trp |
| SGT | 74.6 | IRIS_313-7924 | Bolivia | GJ-trp |
| SGT | 75.0 | IRIS_313-11563 | Nepal | XI-2 |
| SGT | 82.5 | B068 | China | GJ-tmp |
| SGT | 74.3 | IRIS_313-9969 | Sri Lanka | XI-adm |
| SGT | 77.5 | IRIS_313-12269 | Myanmar | XI-adm |
| SGT | 70.8 | IRIS_313-11132 | Myanmar | XI-adm |
| SGT | 76.7 | IRIS_313-7795 | Madagascar | XI-2 |
| SGT | 76.7 | IRIS_313-11741 | Sri Lanka | XI-2 |
| SGT | 70.0 | IRIS_313-12308 | Lao People's Democratic Republic | XI-3 |
| SGT | 73.3 | IRIS_313-11824 | India | XI-2 |
| ASRI | 3.2 | CX132 | India | GJ-trp |
| ASRI | 3.2 | B068 | China | GJ-tmp |
| ASRI | 3.2 | B241 | China | GJ-sbtrp |
| ASRI | 3.1 | IRIS_313-11584 | China | GJ-tmp |
| ASRI | 3.0 | IRIS_313-7838 | Senegal | GJ-trp |
| ASRI | 3.0 | CX315 | North_Korea | GJ-tmp |
| ASRI | 2.9 | IRIS_313-8140 | China | GJ-tmp |
| ASRI | 2.8 | IRIS_313-10543 | India | cA (Aus) |
| ASRI | 2.8 | IRIS_313-11574 | China | GJ-tmp |
| ASRI | 2.8 | IRIS_313-10828 | Philippines | GJ-trp |
| ASRI | 2.8 | IRIS_313-10829 | Philippines | GJ-trp |
| ASRI | 2.8 | B199 | China | GJ-tmp |
| ASRI | 2.8 | IRIS_313-8132 | Portugal | GJ-tmp |
| ASRI | 2.8 | IRIS_313-8481 | China | GJ-tmp |
| ASRI | 2.8 | B056 | China | GJ-tmp |
| ASRI | 2.7 | IRIS_313-11739 | Ghana | GJ-trp |
| ASRI | 2.7 | IRIS_313-10062 | Greece | GJ-trp |

Table S3 Identification of 99 QTLs significantly associated with rice traits related to tolerances to salt and anaerobic stresses at the germination stage by GWAS*.*

| **Population** | **Trait** | **QTL** | **Chr.** | **Peak** | **QTL range** | **P** | **R^2^ (%)** | **MAF** | **Allele** | **Effect** | **Cloned gene** |
| --- | --- | --- | --- | --- | --- | --- | --- | --- | --- | --- | --- |
| G | AGr | *qAGr2* | 2 | 35925631 | 35.00 - 35.9 | 3.00E-05 | 13.1 | 0.02 | C/A | 14.4 |  |
|  | RI | *qRI2* | 2 | 35071007 | 35.00 - 35.10 | 7.00E-08 | 7.7 | 0.48 | T/A | 5.9 |  |
|  | CL | *qCL2* | 2 | 35071007 | 35.00 - 35.10 | 1.00E-07 | 7.6 | 0.48 | T/A | 5.2 |  |
| W | CL | *qCL1* | 1 | 42396181 | 42.30 - 42.51 | 8.00E-07 | 5.6 | 0.04 | C/T | 6.4 | *OsMT2B*  *OrbHLH001* |
|  | RI | *qRI1d* | 1 | 42706181 | 42.70 - 43.05 | 7.00E-07 | 5.7 | 0.04 | C/T | 11.1 |  |
| W | CL | *qCL3b* | 3 | 13769550 | 13.70 - 13.80 | 4.00E-08 | 6.1 | 0.07 | A/G | 6.5 |  |
|  | RI | *qRI3a* | 3 | 13769550 | 13.70 - 13.80 | 2.00E-08 | 6.2 | 0.07 | A/G | 6.5 |  |
| G | CL | *qCL3c* | 3 | 13957011 | 13.30 - 13.90 | 4.00E-06 | 7.9 | 0.02 | C/T | 7.5 |  |
|  | RI | *qRI3b* | 3 | 13957011 | 13.60 - 14.00 | 3.00E-06 | 8 | 0.02 | C/T | 7.5 |  |
| W | CL | *qCL3e* | 3 | 30112801 | 30.00 - 30.40 | 6.00E-07 | 6.1 | 0.27 | T/C | 6.9 |  |
|  | RI | *qRI3c* | 3 | 30342249 | 30.00 - 30.40 | 4.00E-07 | 6.9 | 0.27 | T/C | 7.9 |  |
| X | CL | *qCL4a* | 4 | 16685167 | 16.20 - 16.60 | 6.00E-06 | 7.1 | 0.04 | G/A | -3.9 |  |
|  | RI | *qRI4b* | 4 | 16685167 | 16.50 - 16.90 | 2.00E-06 | 4.4 | 0.04 | G/A | -5.2 |  |
| G+W | CL | *qCL4b* | 4 | 20462761 | 20.30 - 20.50 | 1.00E-07 | 6.4 | 0.06 | G/C | 6.5 |  |
|  | RI | *qRI4c* | 4 | 20462761 | 20.30 - 20.40 | 6.00E-08 | 6.5 | 0.06 | G/C | 6.6 |  |
| G | CL | *qCL4c* | 4 | 31441441 | 31.40 - 31.90 | 2.00E-05 | 6.6 | 0.49 | A/C | 4.5 |  |
|  | RI | *qRI4d* | 4 | 31658984 | 31.60 - 32.74 | 1.00E-05 | 7.5 | 0.49 | A/C | 5.3 |  |
| G+W | RI | *qRI7a* | 7 | 19545842 | 19.50 - 19.60 | 1.00E-05 | 3.6 | 0.06 | T/G | 4.7 |  |
|  | RI | *qRI7b* | 7 | 19614306 | 19.50 - 19.60 | 7.00E-07 | 8.9 | 0.07 | A/G | 4.9 |  |
|  | CL | *qCL7b* | 7 | 19545842 | 19.50 - 19.60 | 7.00E-06 | 3.9 | 0.06 | T/G | 4.8 |  |
| W | CL | *qCL7c* | 7 | 26301442 | 26.20 - 26.40 | 2.00E-08 | 6 | 0.09 | G/A | 6.6 |  |
|  | RI | *qRI7d* | 7 | 26301442 | 26.20 - 26.40 | 1.00E-08 | 6.1 | 0.09 | G/A | 6.9 |  |
| W | CL | *qCL8* | 8 | 18142059 | 18.00 - 18.10 | 7.00E-07 | 6.9 | 0.22 | T/A | 5.1 |  |
|  | RI | *qRI8* | 8 | 18110796 | 18.00 - 18.20 | 1.00E-07 | 5.6 | 0.12 | A/G | 5.8 |  |
| G | CL | *qCL9a* | 9 | 498342 | 0.04 - 0.81 | 7.00E-06 | 8.2 | 0.02 | T/A | 7.8 |  |
|  | RI | *qRI9a* | 9 | 462994 | 0.04 - 0.81 | 4.00E-06 | 8.3 | 0.02 | G/A | 7.9 |  |
| X | AGr | *qAGr9* | 9 | 12283166 | 12.20 - 12.43 | 8.00E-06 | 17.9 | 0.31 | G/A | 12.5 | *OsTPP7* |
|  | CL | *qCL9b* | 9 | 12249903 | 12.21 - 12.40 | 9.00E-07 | 12.3 | 0.44 | G/C | 10.6 |  |
|  | RI | *qRI9b* | 9 | 12249903 | 12.24 - 12.44 | 5.00E-06 | 12.3 | 0.44 | G/C | 9.8 |  |
| W | CL | *qCL10* | 10 | 10626128 | 10.59 - 10.79 | 3.00E-06 | 15.3 | 0.06 | G/C | 10.2 |  |
|  | RI | *qRI10* | 10 | 10605325 | 10.59 - 10.79 | 2.00E-07 | 3.4 | 0.11 | T/C | 12.6 |  |
| X | RI | *qRI11c* | 11 | 22924870 | 22.70 - 22.90 | 8.00E-06 | 8.8 | 0.34 | A/C | 5.6 |  |
| X+W | AGr | *qAGr11b* | 11 | 22973569 | 22.90 - 23.13 | 5.00E-07 | 17.1 | 0.06 | T/C | 9.8 |  |
| W | AGr | *qAGr4* | 4 | 34736543 | 34.60-34.83 | 1.00E-06 | 15.7 | 0.24 | A/G | 12.8 |  |
| G | AGr | *qAGr5* | 5 | 981955 | 0.96 - 10.60 | 5.00E-05 | 14.5 | 0.02 | G/T | -15.2 |  |
| G | AGr | *qAGr8a* | 8 | 9769900 | 9.76 - 9.77 | 2.00E-05 | 12.8 | 0.03 | A/T | 13.9 |  |
| G | AGr | *qAGr8b* | 8 | 15674817 | 14.40 - 15.60 | 2.00E-05 | 17.9 | 0.02 | C/A | 19.5 | *OsCCC1* |
| X | AGr | *qAGr11a* | 11 | 8863237 | 8.30 - 8.80 | 9.00E-06 | 9.4 | 0.17 | C/T | 7.1 |  |
| X | AGr | *qAGr11c* | 11 | 24332736 | 24.30 - 24.70 | 7.00E-06 | 12.8 | 0.13 | A/G | -13.5 |  |
| G | CL | *qCL3a* | 3 | 8402207 | 8.26 - 8.36 | 9.00E-05 | 8.5 | 0.04 | T/C | 5.9 |  |
| G | CL | *qCL3d* | 3 | 28788219 | 28.00 - 29.96 | 6.00E-05 | 5.5 | 0.03 | A/G | -3.3 |  |
| G | CL | *qCL6a* | 6 | 8241111 | 8.03 - 8.30 | 9.00E-05 | 5.6 | 0.02 | C/T | 6.9 |  |
| G | CL | *qCL6b* | 6 | 29473237 | 29.30 - 29.50 | 9.00E-05 | 5.7 | 0.02 | G/T | 4.3 | *OsVHA*-A |
| G | RI | *qRI1a* | 1 | 3198891 | 2.57 - 3.36 | 5.00E-06 | 8.2 | 0.05 | G/C | 7.9 |  |
| G | RI | *qRI1b* | 1 | 6089280 | 6.05 - 7.01 | 8.00E-05 | 8.6 | 0.02 | C/T | 6.8 | *OsGSK1* |
| W | RI | *qRI1c* | 1 | 32673555 | 32.60 - 33.03 | 6.00E-06 | 6.4 | 3267 | G/C | 5.3 | *OsKAT1* |
| G | RI | *qRI4a* | 4 | 1841111 | 1.84 - 2.030 | 6.00E-05 | 5.1 | 0.37 | T/C | 3.4 |  |
| G | RI | *qRI6* | 6 | 25906060 | 25.90 - 26.50 | 4.00E-06 | 5.2 | 0.32 | T/C | 2.8 |  |
| G | RI | *qRI7c* | 7 | 21167800 | 21.10 - 21.40 | 5.00E-05 | 6.1 | 0.05 | G/C | 7.9 |  |
| G | RI | *qRI11a* | 11 | 5476475 | 5.08 - 5.71 | 4.00E-05 | 5.2 | 0.05 | G/T | 2.3 |  |
| G | RI | *qRI11b* | 11 | 5494978 | 5.47 - 5.49 | 7.00E-05 | 6 | 0.04 | C/T | 2.4 |  |
| G | RI | *qRI12a* | 12 | 16559320 | 16.10 - 16.70 | 1.00E-06 | 7.7 | 0.1 | C/G | 2.3 |  |
| G | RI | *qRI12b* | 12 | 19778458 | 19.70 - 19.80 | 6.00E-06 | 16.8 | 0.28 | A/T | -16.3 | *osatg10b* |
| G | RI | *qRI12c* | 12 | 20002699 | 20.80 - 21.00 | 1.00E-05 | 7.5 | 0.12 | A/G | 3.3 |  |
| X+W | BM | *qBM1a* | 1 | 20607675 | 20.60 - 21.10 | 1.00E-07 | 7 | 0.19 | T/C | 7.1 |  |
| G | BM | *qBM1b* | 1 | 40428326 | 40.30 - 40.60 | 5.00E-06 | 6.9 | 0.1 | C/T | 8.1 |  |
| G | BM | *qBM4* | 4 | 17477938 | 17.30 - 17.40 | 3.00E-05 | 6 | 0.04 | A/T | 6.6 |  |
| G | BM | *qBM6a* | 6 | 24370018 | 24.30 - 24.40 | 5.00E-05 | 6.8 | 0.06 | A/G | 8.1 |  |
| X | BM | *qBM6b* | 6 | 27079343 | 27.10 - 27.40 | 1.00E-06 | 5.9 | 0.04 | C/G | -3.2 |  |
| W | BM | *qBM9a* | 9 | 18883921 | 18.82 - 19.14 | 7.00E-08 | 16.1 | 0.29 | G/T | 9.6 | *OsEATB* |
| G | BM | *qBM9b* | 9 | 19006573 | 18.90 - 20.00 | 1.00E-05 | 6.7 | 0.1 | T/C | 5.1 |  |
| G | BM | *qBM10* | 10 | 8290852 | 8.29 - 8.49 | 3.00E-06 | 9.3 | 0.16 | T/A | 8.7 |  |
| X | BM | *qBM12* | 12 | 17682041 | 17.60 - 18.20 | 3.00E-06 | 8 | 0.19 | G/C | 6.1 |  |
| X | SGr | *qSGr3* | 3 | 7151350 | 6.83 - 7.19 | 9.00E-06 | 19.8 | 0.05 | C/T | 10.4 | *dsm3* |
| X | SGr | *qSGr4a* | 4 | 30867945 | 30.70 - 30.90 | 4.00E-07 | 9.2 | 0.05 | G/A | 4.7 |  |
| G | SGr | *qSGr4b* | 4 | 35184540 | 35.10 - 35.30 | 3.00E-05 | 16.1 | 0.36 | C/T | 20.2 |  |
| G | SGr | *qSGr5* | 5 | 19411407 | 19.20 - 19.70 | 4.00E-05 | 16.8 | 0.02 | G/A | 19.9 | *SERF1* |
| X+W | SGr | *qSGr9a* | 9 | 6874417 | 6.82 - 7.05 | 7.00E-06 | 17.2 | 0.11 | G/C | 12.5 | *OsbZIP71* |
| X | SGr | *qSGr9b* | 9 | 21598071 | 21.50 - 21.60 | 1.00E-06 | 11.2 | 0.05 | T/C | 13.3 |  |
| G | SGr | *qSGr10a* | 10 | 5492669 | 5.40 - 6.29 | 5.00E-05 | 11 | 0.2 | T/G | 12.3 |  |
| W | SGr | *qSGr10b* | 10 | 8570783 | 8.38 - 8.58 | 7.00E-06 | 9 | 0.06 | A/G | 6 |  |
| X | SGr | *qSGr10c* | 10 | 18401970 | 18.30 - 18.40 | 9.00E-06 | 8.3 | 0.15 | C/T | 6.8 |  |
| W | SGr | *qSGr10d* | 10 | 22650248 | 22.60 - 22.70 | 1.00E-07 | 12.7 | 0.11 | C/T | 14.9 |  |
| X | SGr | *qSGr11* | 11 | 24969283 | 24.90 - 25.10 | 4.00E-06 | 12 | 0.07 | C/T | 8.1 |  |
| G | SGr | *qSGr12* | 12 | 19781858 | 19.68-19.84 | 3.00E-06 | 8 | 0.02 | A/C | 8.8 | *osatg10b* |
| X+W | ASRI | *qASRI1a* | 1 | 5474339 | 5.47 - 5.38 | 8.00E-05 | 4.5 | 0.04 | C/T | 5.5 |  |
| W | ASRI | *qASRI1b* | 1 | 26882782 | 26.80 - 27.00 | 2.00E-05 | 7.8 | 0.29 | G/T | 5.4 |  |
| X | ASRI | *qASRI1c* | 1 | 32063539 | 32.00 - 33.10 | 4.00E-05 | 3.2 | 0.09 | T/C | 8.5 |  |
| X+W | ASRI | *qASRI1d* | 1 | 34495887 | 34.40 - 35.00 | 2.00E-05 | 8.3 | 0.29 | T/C | 5.4 | *OsHsfA7* |
| X | ASRI | *qASRI1e* | 1 | 39785476 | 39.70 - 40.10 | 1.00E-04 | 2.8 | 0.44 | C/T | -4.8 | *Osabf1* |
| W | ASRI | *qASRI2a* | 2 | 5557603 | 5.55 - 6.90 | 7.00E-05 | 10.2 | 0.34 | C/T | 2.7 |  |
| X | ASRI | *qASRI2b* | 2 | 7803763 | 7.80 - 8.10 | 1.00E-04 | 3.4 | 0.35 | A/G | 5.5 |  |
| W | ASRI | *qASRI2c* | 2 | 8050121 | 8.04 - 8.42 | 9.00E-06 | 5.7 | 0.05 | G/A | -4.5 |  |
| G | ASRI | *qASRI2d* | 2 | 9891728 | 9.89 - 10.10 | 7.00E-05 | 3.1 | 0.02 | C/A | 4.5 | *OsGMST1* |
| X+W | ASRI | *qASRI2e* | 2 | 18780404 | 18.70 - 19.10 | 1.00E-05 | 7.3 | 0.05 | T/C | 5.5 | *OsGRX8* |
| W | ASRI | *qASRI2f* | 2 | 23616491 | 23.60 - 23.90 | 8.00E-05 | 10.1 | 0.49 | C/T | 5.8 | rss1 |
| X+W | ASRI | *qASRI2g* | 2 | 34798618 | 34.70 - 35.10 | 9.00E-05 | 7.1 | 0.42 | C/G | 10.2 | *OsbZIP23* |
| W | ASRI | *qASRI3a* | 3 | 2533049 | 2.53 - 2.99 | 9.00E-05 | 10.2 | 0.09 | A/G | 5.9 |  |
| W | ASRI | *qASRI3b* | 3 | 5991519 | 5.99 - 6.10 | 1.00E-05 | 3.4 | 0.35 | C/T | -4.1 |  |
| W | ASRI | *qASRI3c* | 3 | 19721975 | 19.70 - 20.10 | 8.00E-05 | 4.3 | 0.22 | A/G | -2.9 |  |
| W | ASRI | *qASRI3d* | 3 | 20635449 | 20.60 - 21.10 | 1.00E-04 | 6.2 | 0.29 | G/A | 2.9 |  |
| X+W | ASRI | *qASRI3e* | 3 | 26782090 | 26.70 - 26.80 | 2.00E-05 | 9.2 | 0.21 | G/A | 11.3 |  |
| X | ASRI | *qASRI3f* | 3 | 34371331 | 33.50-34.8 | 6.00E-05 | 3.1 | 0.31 | A/G | -3.2 | DST |
| X+W+G | ASRI | *qASRI4a* | 4 | 4269101 | 4.26 - 4.29 | 9.00E-05 | 9.3 | 0.06 | A/G | 8.5 |  |
| G | ASRI | *qASRI4b* | 4 | 17766112 | 17.70 - 18.10 | 9.00E-05 | 4.1 | 0.02 | C/A | 6.6 |  |
| X | ASRI | *qASRI4c* | 4 | 28008438 | 28.00 - 39.10 | 2.00E-05 | 4.1 | 0.02 | A/G | 5.6 | *OsCPK12* |
| G+W | ASRI | *qASRI4d* | 4 | 30463511 | 30.40 - 31.10 | 4.00E-05 | 5.3 | 0.03 | G/A | 7.5 |  |
| X | ASRI | *qASRI4e* | 4 | 33496910 | 33.40 - 33.50 | 9.00E-05 | 4 | 0.08 | G/A | 5.5 |  |
| W | ASRI | *qASRI5a* | 5 | 1028111 | 1.02 - 2.10 | 8.00E-05 | 8 | 0.1 | C/T | 3.4 |  |
| W | ASRI | *qASRI5b* | 5 | 22094216 | 22.00 - 23.10 | 2.00E-05 | 3.5 | 0.29 | T/C | -2.1 |  |
| W | ASRI | *qASRI5c* | 5 | 26518017 | 26.50 - 26.90 | 3.00E-05 | 3.5 | 0.35 | G/A | -3.1 |  |
| G | ASRI | *qASRI6a* | 6 | 9343925 | 9.34 - 10.10 | 6.00E-05 | 2.7 | 0.32 | T/G | 4.6 |  |
| W | ASRI | *qASRI6b* | 6 | 10017648 | 13.10 - 13.70 | 6.00E-05 | 2.8 | 0.43 | G/A | 2.9 |  |
| W | ASRI | *qASRI6c* | 6 | 27636000 | 27.60 - 28.10 | 8.00E-05 | 8.2 | 0.1 | A/G | 4 |  |
| X+W+G | ASRI | *qASRI6d* | 6 | 30832099 | 30.80 - 31.10 | 2.00E-05 | 4 | 0.07 | A/T | 9.5 |  |
| W | ASRI | *qASRI7a* | 7 | 7223570 | 7.22 - 7.50 | 8.00E-05 | 5.3 | 0.36 | T/G | -9.7 |  |
| W | ASRI | *qASRI7b* | 7 | 24617262 | 24.60 - 25.10 | 6.00E-05 | 3.3 | 0.35 | G/A | -9.9 |  |
| W | ASRI | *qASRI8* | 8 | 27461158 | 27.40 - 27.60 | 2.00E-05 | 5.5 | 0.26 | C/T | 7 |  |
| W | ASRI | *qASRI9* | 9 | 21335551 | 21.30 - 22.10 | 9.00E-05 | 5.3 | 0.04 | C/G | 4.5 |  |
| W | ASRI | *qASRI10a* | 10 | 3054964 | 3.05 - 4.99 | 1.00E-05 | 8.1 | 0.26 | G/A | 3.8 |  |
| W | ASRI | *qASRI10b* | 10 | 5164205 | 5.16 - 6.48 | 5.00E-05 | 7.3 | 0.17 | C/T | 6.5 |  |
| W | ASRI | *qASRI10c* | 10 | 10224177 | 10.20 - 11.10 | 1.00E-04 | 6.3 | 0.04 | G/C | 5 |  |
| G+W | ASRI | *qASRI11a* | 11 | 4433184 | 4.43 - 4.59 | 3.00E-05 | 4.3 | 0.1 | C/T | 8 |  |
| G+W | ASRI | *qASRI11b* | 11 | 22499596 | 22.40 - 22.50 | 2.00E-05 | 10.2 | 0.07 | G/A | 7 |  |
| X+W | ASRI | *qASRI12a* | 12 | 143367 | 0.14 - 1.10 | 3.00E-05 | 8.3 | 0.06 | G/A | 6.5 |  |
| X+W | ASRI | *qASRI12b* | 12 | 4499779 | 4.49 - 5.10 | 8.00E-05 | 5.3 | 0.32 | T/C | 9.9 |  |
| X | ASRI | *qASRI12c* | 12 | 7596998 | 7.59 - 8.10 | 4.00E-05 | 3.1 | 0.34 | C/T | -2.1 |  |
